# A New Duplex PCR-Assay for the Detection and Identification of *Paracoccidioides* Species

**DOI:** 10.3390/jof7030169

**Published:** 2021-02-26

**Authors:** Breno Gonçalves Pinheiro, Ana Paula Pôssa, Paula Portella Della Terra, Jamile Ambrósio de Carvalho, Giannina Ricci, Angela Satie Nishikaku, Rosane Christine Hahn, Zoilo Pires de Camargo, Anderson Messias Rodrigues

**Affiliations:** 1 Laboratory of Emerging Fungal Pathogens, Department of Microbiology, Immunology, and Parasitology, Discipline of Cellular Biology, Federal University of São Paulo (UNIFESP), São Paulo 04023062, Brazil; brenogonpi@gmail.com (B.G.P.); anappossa@gmail.com (A.P.P.); pauladellaterra@gmail.com (P.P.D.T.); jamileambrosio@hotmail.com (J.A.d.C.); zpcamargo1@gmail.com (Z.P.d.C.); 2 Department of Medicine, Discipline of infectious Diseases, Federal University of São Paulo (UNIFESP), São Paulo 04023062, Brazil; 3 Centro de Diagnóstico e Pesquisa em Biologia Molecular Dr. Ivo Ricci, São Paulo 13561020, Brazil; gianninaricci@uol.com.br (G.R.); angelanishikaku@yahoo.com (A.S.N.); 4 Laboratory of Mycology/Research, Faculty of Medicine, Federal University of Mato Grosso, Cuiabá 78060900, Brazil; rchahn@terra.com.br; 5 Júlio Muller University Hospital, Federal University of Mato Grosso, Cuiabá 78048902, Brazil

**Keywords:** molecular diagnostics, species-specific PCR, duplex PCR, *Paracoccidioides brasiliensis*, *Paracoccidioides lutzii*, paracoccidioidomycosis, epidemiology, diagnosis, FFPE, systemic mycosis

## Abstract

Paracoccidioidomycosis (PCM) is a life-threatening systemic fungal infection caused by members of the *Paracoccidioides brasiliensis* complex and *P. lutzii*. Routine diagnoses of PCM down to the species level using classical mycological approaches are unspecific due to overlapping phenotypes. There is an urgent need for specific, sensitive, and cost-effective molecular tools to diagnose PCM. Variation among the exon-2 of the gp43 gene was exploited to design species-specific primer pairs to discriminate between members of the *P. brasiliensis* complex and *P. lutzii* in a duplex PCR assay. Primer-BLAST searches revealed highly species-specific primers, and no significant region of homology was found against DNA databases except for *Paracoccidioides* species. Primers PbraCx-F and PbraCx-R targeting *P. brasiliensis* DNA produced an amplicon of 308 bp, while primers Plu-F and Plu-R targeting *P. lutzii* DNA generated an amplicon of 142 bp. The lower limit of detection for our duplex PCR assay was 1 pg of gDNA. A panel of 62 *Paracoccidioides* revealed 100% specificity (AUC = 1.000, 95%CI 0.972–1.000, *p* < 0.0001) without cross-reacting with other medically relevant fungi or human DNA. As a proof of concept, we demonstrated the accurate identification of the *P. brasiliensis* complex (*n* = 7) or *P. lutzii* (*n* = 6) from a broad range of formalin-fixed, paraffin-embedded (FFPE) tissues of PCM patient’s organs. In four cases, FFPE PCR results confirmed, for the first time, co-infection due to *P. brasiliensis* (S1) and *P. lutzii* in the same biopsy. Our duplex PCR assay is useful to detect and differentiate members of the *P. brasiliensis* complex and *P. lutzii*, providing clinical laboratories with an important tool to be applied routinely, especially in atypical cases such as those featuring negative serology and positive mycological examination of clinical specimens as well as for the investigation of putative co-infection cases. This will likely benefit thousands of infected patients every year in a wide area of the Americas.

## 1. Introduction

Paracoccidioidomycosis (PCM) is a systemic mycosis affecting the lungs, skin, mucous membranes, lymph nodes, and internal organs, first described by Adolfo Lutz in 1908 [1,2]. Breathing in soilborne fungal propagules can lead to primary pulmonary infection in humans. Therefore, the risk of developing PCM is generally greater for people with exposure to soil-disturbing activities such as agriculture, excavation, soil preparation, gardening, and transportation of vegetable products [3]. Patients can develop a broad spectrum of clinical manifestations, ranging from a mild illness and localized condition to a more severe and disseminated PCM, depending on the extent of the depression of cellular immunity [3,4,5,6]. Two major clinical manifestations are an acute or subacute form (also known as juvenile), which is prevalent in children and young adults [7], and a unifocal or multifocal chronic form, which is most prevalent in adult males and has predominant pulmonary and/or mucocutaneous involvement [8].

Since the 1930s, mycologists have used the term *Paracoccidioides brasiliensis* (Splendore 1912) to refer to the PCM agent [9], and for over 80 years, *Paracoccidioides* was considered a monotypic taxon. Epidemiological studies associated with classical DNA fingerprinting methods, such as restriction fragment length polymorphism (RFLP) [10], random amplification of polymorphic DNA (RAPD) [11,12], and short tandem repeat (STR) [13], revealed a remarkable genetic diversity among clinical and environmental isolates of *Paracoccidioides* [14,15,16], thus supporting the existence of cryptic species. Multilocus sequence analysis of protein-coding loci (e.g., the chitin synthase, β-glucan synthase, α-tubulin, adenyl ribosylation factor, and PbGP43 gene) confirmed that *Paracoccidioides brasiliensis* consists of a complex of at least four distinct phylogenetic species, including the genetic groups S1 (species 1), PS2 (phylogenetic species 2), and PS3 (phylogenetic species 3) reported by Matute et al. [17], in addition to PS4 (phylogenetic species 4), recognized by Teixeira et al. [18]. Interestingly, isolates recovered from Brazil’s central and northern regions are identified as belonging to a divergent genotype, initially referred to as Pb01-like and more recently called *P. lutzii* [19].

Turissini et al. [20] combined nuclear and mitochondrial markers to evaluate species boundaries in members of the *P. brasiliensis* complex and associated genetic diversity with speciation, proposing to elevate the cryptic groups to the species level. Consequently, the S1 group now corresponds to *P. brasiliensis s. str.* and is by far the most common human PCM agent recovered from Argentina, Brazil, Paraguay, Peru, and Venezuela. The remaining groups are unevenly distributed. For example, PS2 (named *P. americana*) has been reported in clinical and animal cases in Brazil and Venezuela. The PS3 group, called *P. restrepiensis*, consists of a monophyletic clade and clonal population that has been recovered in humans and armadillos in the endemic regions of Colombia, Brazil, Peru, and Argentina [18,21,22,23]. The PS4 clade is now named *P. venezuelensis* in reference to the geographically restricted area where the clinical isolates originated [24]. *Paracoccidioides lutzii* mycosis’s epicenter occurs in Brazil’s central-western region, although a few cases have been reported from remote areas [19,25,26,27]. However, the significance of taxonomic changes in PCM’s clinical practice still needs to be investigated in depth [5,28,29].

The diagnosis of PCM is based on clinical and biological information. The laboratory investigation includes direct mycological research, histopathological examination, and isolation of the agent in culture [30]. The presence of pathognomonic birefringent yeast cells allows a generic rather than species identification of *Paracoccidioides*. In *Paracoccidioides*, a reliable identification directly from clinical specimens using exclusively morphological features is unfeasible due to the overlap in yeast morphology (the parasitic phase) [30]. The phenotypic recognition problem exists even when using microorganism cultures in mycelial growth due to the limited number of discernible morphological traits to differentiate members of the *P. brasiliensis* complex and *P. lutzii*.

In this scenario, molecular techniques have the potential to greatly facilitate the recognition of *Paracoccidioides*. DNA sequencing followed by phylogenetic analysis is the reference method for species recognition [15,17,18,24,31]. A large range of methods based on polymerase chain reaction (PCR), such as PCR-RFLP (restriction fragment length polymorphism) [32], nested PCR [33], quantitative real-time PCR [24], and multilocus microsatellite typing (MLMT) [13], have been applied to complement microbiological diagnosis. Unfortunately, none of these methods can discriminate among the five taxa described to date with certainty. Therefore, discrimination of the *P. brasiliensis* complex and *P. lutzii* is the only secure strategy to diagnose PCM [34].

This study aimed to develop a robust, accurate, and cost-effective species-specific duplex PCR-based assay capable of detecting and differentiating members of the *P. brasiliensis* complex and *P. lutzii*, providing clinical laboratories with an important tool to be routinely applied, especially in atypical cases such as those involving patients with negative serology and positive mycological examination of clinical specimens as well as for the investigation of putative co-infection cases. This will likely benefit thousands of infected patients every year in a wide area of the Americas.

## 2. Materials and Methods

### 2.1. Ethical Approval

This study was approved by the Ethics in Research Committee of the Federal University of São Paulo under protocol numbers 9771060120 and 3147220120.

### 2.2. Fungal Strains and Culture Conditions

Clinical and environmental *Paracoccidioides* spp. isolates were obtained from the culture collection of the Federal University of São Paulo (UNIFESP), São Paulo, Brazil. Isolates were kept as slants on Fava-Netto medium [35,36] at 37 °C in yeast form. These isolates were previously characterized down to species level by *TUB1*-RFLP as earlier described by Roberto et al. [32]. Reference strains representing the main *Paracoccidioides* species covering a plethora of genotypes were included in all experiments (Supplementary Table S1).

### 2.3. DNA Extraction

DNA was extracted and purified directly from 10-day old yeast colonies using the Fast DNA kit (MP Biomedicals, Vista, CA, USA) according to the manufacturer’s instructions. DNA concentration and purity were determined using a NanoDrop 2000 spectrophotometer (Thermo Fisher Scientific, Waltham, MA, USA). Subsequently, DNA was diluted to a final concentration of 100 ng/μL and stored in a freezer at −20 °C until use. For the assessment of quality control of the whole extraction process, *Paracoccidioides* DNAs were used in PCRs with the universal primers ITS1 (5′-TCC GTA GGT GAA CCT TGC GG) and ITS4 (5′-TCC TCC GCT TAT TGA TAT GC) [37], targeting the ribosomal DNA operon. Amplicons were resolved by 1.2% agarose gel electrophoresis, and amplification of a single product using ITS1/ITS4 primers was regarded as a sample free of PCR inhibitors.

### 2.4. Primer Design

Reference sequences in the present study included the gp43 nucleotide sequences covering exon 2 from 82 isolates identified as *Paracoccidioides* spp. We also included gp43 sequences from related species, genera, and other pathogens (e.g., *Lacazia loboi*) to increase our dataset’s genetic diversity and cover most genotypes described to date in the literature. These sequences were previously deposited in the GenBank and described by Matute et al. [17], Teixeira et al. [19], Hahn et al. [38], Marques-da-Silva et al. [26], and Vilela et al. [39] (Supplementary Table S2). Sequences retrieved from the GenBank were aligned with MAFFT 7 [40]. Alignments of the PbGP43 gene sequence were corrected manually with the MEGA 7 software [41] to avoid mispairing bases. Gp43 sequences were used to identify parsimony informative sites that were either conserved or divergent intraspecifically, which could be used for primer design to differentiate the *P. brasiliensis* complex (i.e., S1, PS2, PS3, and PS4) and *P. lutzii*. We chose short, informative regions with variations of 18–25 nucleotides based on nucleotide polymorphisms. The software Primer3 [42] (http://primer3.wi.mit.edu/; Accessed: 21 August 2018) was used to evaluate melting temperatures, %CG contents, dimer sequences, and mismatches in candidate sequences. Next, candidate primers were evaluated with the Mfold software [43] for potential secondary structures, which could reduce amplification efficiency. Finally, we selected primers that would generate different product sizes for distinct species to facilitate identification with gel electrophoresis in duplex assays. Candidate primers were evaluated in vitro with conventional PCR, followed by gel electrophoresis and UV detection.

### 2.5. In Silico PCR

In silico PCR was performed to validate each primer pair’s specificity in detecting the DNA of *Paracoccidioides* species using the Primer-BLAST program [44], available at http://www.ncbi.nlm.nih.gov/tools/primer-blast/ (Accessed: 15 April 2019). Primer specificity was initially evaluated in silico to screen unintended targets from genomic databases, included Ref-Seq mRNA, Genome (reference assembly from selected organisms and chromosomes from all organisms), and non-redundant databases, including Eukarya (taxid:2759), Fungi (taxid:4751), Bacteria (taxid:2), and Viruses (taxid:10239). Primer specificity stringency was set to recognize sequences with at least two total mismatches, to identify unintended similar targets, and at least two mismatches had to appear within the last 5 bps at the 3’ end. Targets that had four or more mismatches with a single primer were ignored [44].

### 2.6. PCR Optimization and Gel Electrophoresis

Total DNA extracted from *Paracoccidioides* species was directly used as a PCR template for each candidate species-specific primer pair evaluated. The PCRs were performed in a final volume of 25 μL, including 12.5 μL of PCR Master Mix buffer (2×), which contained 3 mM MgCl_2_, 400 mM of each dNTP, and 50 U/mL of Taq polymerase (Promega Corporation, Madison, WI, USA); 7.5 μL of water, 1 μL of each of forward and reverse primer (10 pmol/μL; Integrated DNA Technologies, Coralville, IA, USA), and 1 μL of target DNA [100 ng/μL]. PCRs were performed in an Eppendorf Mastercycler Pro system (Eppendorf, Hamburg, Germany). We used the touchdown PCR method to improve the specificity during amplification. The conditions were as follows: an initial denaturing step of 5 min at 95 °C, followed by 35 cycles of 1 min at 95 °C, 1 min at the annealing temperature (touchdown PCR), and 1 min at 72 °C, and a final step of 10 min at 72 °C. In the touchdown protocol, the annealing temperature in the first cycle was 70 °C, after which the annealing temperature was reduced by 1 °C/2 cycles for the next 20 cycles. Finally, the PCR was completed with an annealing temperature of 60 °C for the remaining 15 cycles [45]. Electrophoresis of amplicons was carried out in the presence of GelRed (Biotium, Hayward, CA, USA) with 1.2% agarose gel in 1× TBE buffer at 100 V for 1 h at room temperature. Gels were imaged under UV light with the L-Pix Touch imaging system (Loccus Biotecnologia, São Paulo, Brazil).

### 2.7. Assay Specificity

For forward and reverse primers pairs, we evaluated specificity with PCR amplification of DNA samples derived from clinical and environmental representatives of Onygenales and related species. In total, we tested 68 DNA samples derived from other pathogenic fungi, including agents of superficial, subcutaneous, and systemic mycoses in humans and animals. The PCR conditions and gel electrophoresis were as described above.

Human DNA samples from A549 cells, a cell line derived from a pulmonary adenocarcinoma, were included as specificity controls [46]. Human DNA was extracted and quantified as described earlier using the Fast DNA kit with CLS-TS reagent [45]. As control of DNA quality, A549 samples were tested by PCR using a quadruplex-assay targeting nonoverlapping sites in the GAPDH gene (chr12) [47]. Samples that produced 100, 200, 300, and 400 bp fragments were regarded to be free of PCR inhibitors. A549 cell DNA (50 ng) was spiked or not with an equal amount of *Paracoccidioides* DNA and used in the duplex assay to confirm our primers’ absence of cross-reactivity with human DNA.

### 2.8. Assay Sensitivity

We evaluated the sensitivity to ensure reliable amplification at low levels of the target DNA. We performed 10-fold serial dilutions of the DNA, starting with 100 ng/μL and ending with 0.01 fg/μL. The detection limit was noted for the singleplex and duplex assays.

### 2.9. Non-Target Template Competition

A pooled DNA sample was created by mixing equal volumes of total DNA (50 ng/μL per strain) extracted from *P. brasiliensis* S1 (Pb18), *P. brasiliensis* PS2 (EPM204), *P. brasiliensis* PS3 (EPM77), *P. brasiliensis* PS4 (EPM73), and *P. lutzii* (Pb01). Then, 2 μL of this pool was loaded as a target in duplex PCR reactions, as described above. To confirm the specificity of amplification, we removed the target DNA sample from the pooled mixture (pool) for each primer combination.

### 2.10. Detection of Paracoccidioides DNA from BALB/c Lungs and Soil

To determine the viability of our duplex assay for sensitive and sequence-specific detection of *Paracoccidioides* DNA within a complex mixture containing soil or host nucleic acids, we used artificially contaminated (spiked) environmental and animal samples, respectively. For environmental detection, DNA was extracted from 50–100 mg soil samples by combining lysis of microbial cells via cell lytic buffer (800 µL of CLS-VF and 200 µl of PPS buffer; Fast DNA kit, MP Biomedicals, Vista, CA, USA) and the bead beating method as described earlier [48]. Afterward, we used 1 μL of the environmental DNA samples (100 ng/μL) in our duplex assay along with 10-fold serial dilutions of the *Paracoccidioides* DNA (spiked), starting with 100 ng/μL and ending with 0.01 fg/μL. Negative controls included non-spiked samples. To evaluate the DNA quality, we also tested all samples (spiked and non-spiked) by PCR using universal primers ITS1 and ITS4 as described earlier [37].

For detection from host samples, fresh tissue fragments (~20 mg) from the lungs of non-infected BALB/c mice were placed into 2 mL screw-cap tubes containing a ceramic bead with 0.25” in diameter, matrix A, plus 1 mL CLS-TS (MP Biomedicals, Vista, CA, USA), and co-extracted with *Paracoccidioides* species yeast cells (1 × 10^5^ yeasts), as previously described by our group [45,48]. The DNA quality was assessed by amplifying the β-actin gene in the BALB/c genome, as described by Pahl et al. [49]. A positive β-actin amplification confirmed that the samples were free of PCR inhibitors.

### 2.11. Detection of Paracoccidioides DNA from Formalin-Fixed, Paraffin-Embedded (FFPE) Tissue Sections

Twelve formalin-fixed, paraffin-embedded (FFPE) tissue samples from the tibia (*n* = 2), duodenum (*n* = 1), lungs (*n* = 1), lymph node (*n* = 4), oral mucosa (*n* = 2), and skin (*n* = 2) were obtained from eight patients (13–63 years old) in Brazil (Supplementary Table S3). PCM had been diagnosed by histopathology in all samples. DNA was extracted from three 5-µm thick sections of each sample using the QIAamp DNA FFPE Tissue Kit (Qiagen, Hilden, Germany) following the manufacturer’s instructions [50,51]. All samples were collected in full compliance with regulatory standards and best-practice guidelines [52,53,54]. The isolated DNA was eluted in 70 µl of elution buffer [51], and DNA quality was assessed by PCR using a quadruplex-assay targeting nonoverlapping sites in the GAPDH gene (chr12) [47]. Only samples that amplified at least three bands (e.g., 100, 200, and 300 bp) were considered in our duplex assay for searching *Paracoccidioides* DNA. A volume of 5 µL of FFPE DNA template was added to the duplex PCR assay. PCR cycling conditions were the same as described above (Section 2.6), except that we increased the total number of cycles to 40 instead of 35 cycles, aiming to achieve a greater amount of PCR products.

Amplified products (i.e., 308- or 142-bp specific regions of the gp43) were gel-purified using the Wizard SV Gel and PCR Clean-Up System (Promega, Madison, WI, USA), following the manufacturer’s instructions. The fungal PCR products were control sequenced directly in two reactions with forward and reverse primers to increase sequence data quality (*Phred* ≥ 30). The sequencing reactions were performed with the BigDye Terminator v3.1 Cycle Sequencing Kit (Applied Biosystems, Inc., Foster City, CA, USA), and the sequences were determined with a SeqStudio Genetic Analyzer System. Sequences were assembled into single sequences via CAP3 implemented in BioEdit software [55], aligned with MAFFT v. 7 [40], and retrieved alignments were manually edited to avoid mispaired bases. All sequences >200 bp were deposited online at GenBank (Accession numbers: MW556435 -MW556441).

Genetic relationships and DNA polymorphisms were investigated using DnaSP software version 6 [56]. The nucleotide (π) as well as the haplotype (Hd) diversities [57] were calculated for the FFPE-derived sequences generated by using the 308-bp fragment only. Sites containing gaps and missing data were considered in the analysis. The haplotype network analysis was constructed using the Median-Joining method [57], implemented into the software NETWORK version 5.0 (Fluxus Technology Ltd., Suffolk, UK), as previously described [58].

### 2.12. Statistical Analysis

Sensitivity, specificity, positive predictive value (PPV), and negative predictive value (NPV) were included as diagnostic parameters. Receiver operating characteristic (ROC) curves were plotted and analyzed to determine our duplex assay’s sensitivity in detecting members of the *P. brasiliensis* complex and *P. lutzii*. The area under the curve (AUC) in the ROC analysis was estimated as a test performance measure. ROC values were interpreted as follows: 0.50–0.60, failed; 0.60–0.70, poor; 0.70–0.80, fair; 0.80–0.90, good; and 0.90–1.0, excellent discrimination ability. *p*-values ≤0.05 were considered statistically significant. The concordance of our duplex assay and *TUB1*-RFLP previously reported by Roberto et al. [32] was calculated using the Kappa statistic and its 95% confidence interval (CI). The strength of agreement was inferred as follows: 0.00–0.20, poor agreement; 0.21–0.40, fair agreement; 0.41–0.60, moderate agreement; 0.61–0.80, good agreement; and 0.81–1.00, very good agreement [59]. All calculations were performed with the MedCalc statistical software, version 14.8.1 (MedCalc Software bvba; http://www.medcalc.org accessed on 15 January 2021).

## 3. Results

**Figure 1:**
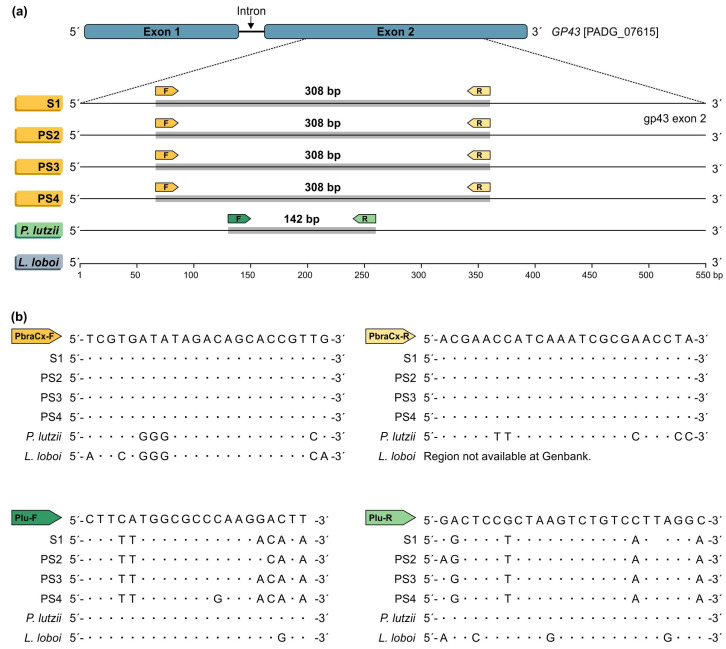
In silico PCR showing homology regions with sequences deposited in the GenBank. (**a**) Primers were designed to match the target gene gp43 exon-2. Primers PbraCx-F and PbraCx-R targeting *P. brasiliensis* complex DNA produced an amplicon with an expected size of 308 bp, while primers Plu-F and Plu-R targeting *P. lutzii* DNA generated an amplicon with 142 bp. (**b**) Interspecific polymorphisms in the primers’ annealing region between *P. brasiliensis* complex isolates and *P. lutzii* or between *Paracoccidioides* and the closely related species *Lacazia loboi* supported the specificity of the primers developed.

Based on the gp43 exon2 partial sequences, the dataset for the primer design of the *Paracoccidioides* isolates comprised 82 sequences covering members of the *P. brasiliensis* complex (*n* = 65) and *P. lutzii* (*n* = 17). The aligned gp43 sequences covered 54.25% of exon 2 and were 427 bp long, including 359 invariable characters, 60 variable parsimony-informative sites (14.05%), and 8 singletons. Inspection of the variable parsimony-informative site regions did not reveal a fully conserved intraspecific stretch with enough length for a candidate PCR primer targeting cryptic species embedded in the *P. brasiliensis* species complex (i.e., *P. brasiliensis s. str.*, *P. americana*, *P. restrepiensis*, and *P. venezuelensis*). Therefore, a strategy was employed to differentiate *P. brasiliensis* complex and *P. lutzii* using a single-round duplex PCR assay. From the best candidate regions, the chosen one is shown in Figure 1, which is highly conserved among all retrieved sequences and corresponds to target regions of the *P. brasiliensis* complex or *P. lutzii*. The primers PbraCx-F and PbraCx-R were developed to selectively amplify members of the *P. brasiliensis* complex, and the primers Plu-F and Plu-R were designed to target *P. lutzii* isolates (Figure 1; Table 1).

In silico analyses using each primer pair sequence with Primer3 [42] and Mfold software [43] confirmed the compatibility of the chosen primer pairs (e.g., no dimer sequences, low potential secondary structures) and provided the optimal annealing temperatures, supporting their use in a duplex reaction. The expected size of the *P. brasiliensis* complex amplicon using PbraCx-F and PbraCx-R primers was 308 bp, whereas Plu-F and Plu-R primers for *P. lutzii* produced an amplicon of 142 bp. Amplicons with deviating sizes were fundamental to choose the best candidate for each *Paracoccidioides* species, facilitating the analysis of the results using conventional gel electrophoresis systems.

BLASTn-searches [44] predicted a highly species-specific assay, and no significant region of homology was found with visited taxa, including Eukarya (taxid:2759), Fungi (taxid:4751), Bacteria (taxid:2), and Viruses (taxid:10239), except the *Paracoccidioides* species. By combining Primer-BLAST to ensure a full primer-target alignment against a rich sequence database available in the GenBank, we demonstrated our assay’s specificity in silico [44] (Supplementary Table S4).

**Figure 2:**
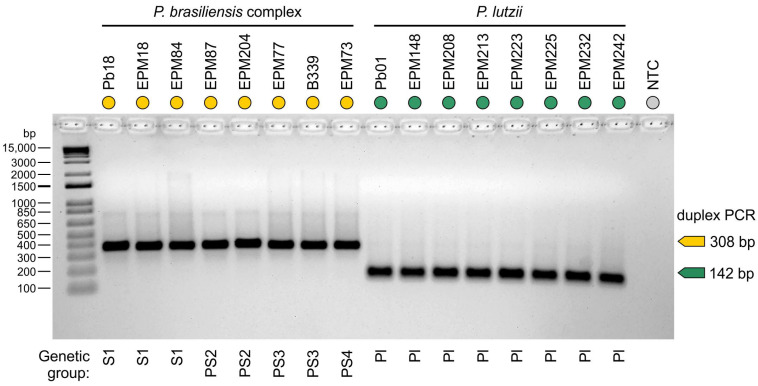
Agarose gel electrophoresis showing successful amplification with specific primer sets targeting members of the *P. brasiliensis* complex (PbraCx-F and PbraCx-R) and *P. lutzii* (Plu-F and Plu-R) in a duplex PCR assay. A significant difference in amplicon sizes encourages the use of agarose gel electrophoresis for resolving amplicons produced for *Paracoccidioides* species. NTC: no template control. Amplicons were control sequenced, and representative sequences >200 bp were deposited online at GenBank (Accession numbers: MW556431–MW556434).

Each *Paracoccidioides* DNA sample used in this study was diluted to 100 ng/μL before the in vitro PCR assay. The tests for *P. brasiliensis* complex isolates (*n* = 49) showed, in each case, that a unique DNA fragment corresponding to the predicted size of 308 bp was resolved after gel electrophoresis, while all *P. lutzii* isolates (*n* = 13) showed a unique amplicon of 142 bp (Figure 2).

To confirm the specificity of our duplex assay, we used 68 isolates representing non-target DNA, including *S. brasiliensis* (*n* = 12), *S. schenckii* (*n* = 12), *S. globosa* (*n* = 8), *S. pallida* (*n* = 1), *S. chilensis* (*n* = 1), *S. mexicana* (*n* = 2), *Histoplasma capsulatum* (*n* = 4), *Aspergillus* spp. (*n* = 1), *Chrysosporium* spp. (*n* = 1), *Debaryomyces* spp. (*n* = 1), *Candida krusei* (*n* = 1), *Candida albicans* (*n* = 1), *Candida parapsilosis* (*n* = 2), *Candida orthopsilosis* (*n* = 2), *Candida metapsilosis* (*n* = 1), *Cryptococcus neoformans* (*n* = 4), *Cryptococcus gattii* (*n* = 4), and *Coccidioides posadasii* (*n* = 10), which did not yield any detectable PCR product. This result supports the high specificity predicted in the BLASTn-search analyses.

**Figure 3:**
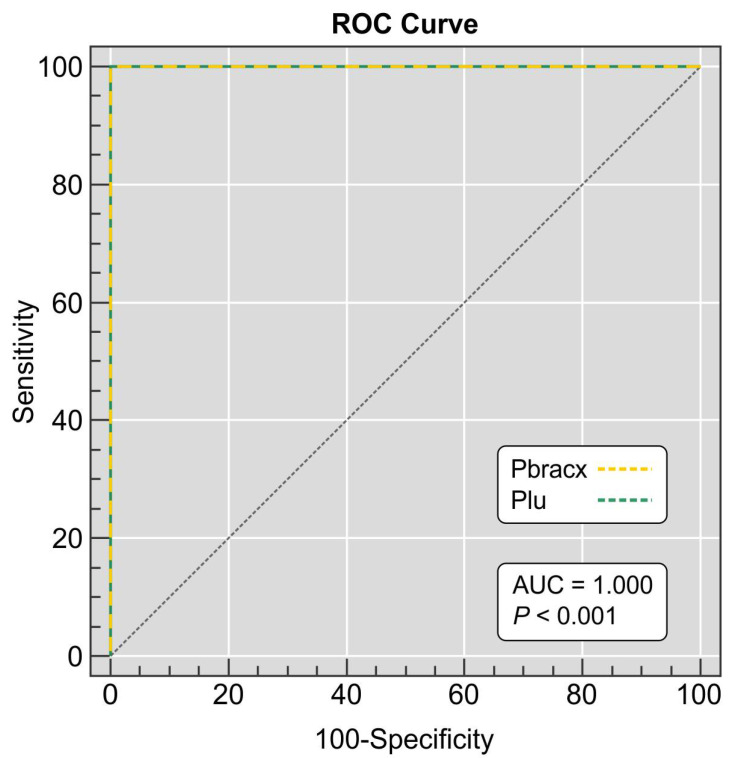
Receiver operating characteristics (ROCs) depicting duplex PCR assay sensitivity and specificity. Samples were from 49 *P. brasiliensis* complex isolates, 13 *P. lutzii* isolates, and 68 non-target fungal DNA derived from agents of superficial, subcutaneous, and systemic mycoses in humans and animals. The ROC is plotted between the true-positive rate (sensitivity) on the *y*-axis and the false-positive rate (100-specificity) on the *x*-axis. The area under the curve (AUC) represents the accuracy of the duplex assay, which was 1.000 (95% CI 0.972–1.000, *p* < 0.0001).

Analysis of the area under the curve (AUC) provided a good indication of the utility of our assay (AUC = 1.000, 95% CI 0.972–1.000, *p* < 0.0001) in differentiating the *P. brasiliensis* complex and *P. lutzii* isolates (Figure 3). Our duplex PCR was in full agreement (100%) with the identifications achieved with molecular phylogeny [5,26,38] and *TUB1*-RFLP (*Kappa* = 1.0; 95% CI = 1.0–1.0; very good agreement) [32] carried out previously at our laboratory, reinforcing the usefulness of our assay to discriminate members of the *P. brasiliensis* complex and *P. lutzii*. All negative controls remained negative after PCR.

**Figure 4:**
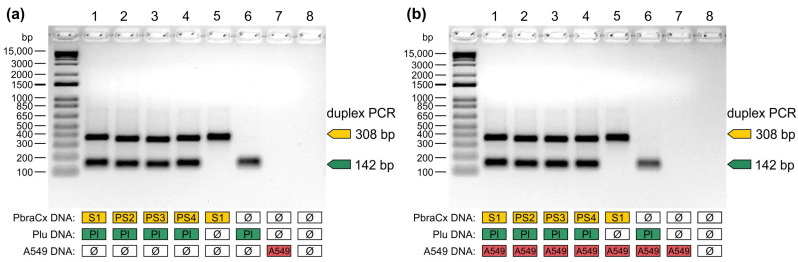
The specificity of *Paracoccidioides* duplex assay in the presence of non-target templates. (**a**) A pooled DNA sample comprising equal amounts of members of the *P. brasiliensis* complex and *P. lutzii*. To confirm the specificity of amplification, we removed the target DNA from the pooled sample. Lane 1a: Pb18 (S1) and Pb01 (*P. lutzii*); Lane 2a: EPM204 (PS2) and Pb01; Lane 3a: EPM77 (PS3) and Pb01; Lane 4a: EPM73 (PS4) and Pb01; Lane 5a: Pb18, EPM204, EPM77, and EPM73; Lane 6a: Pb01; Lane 7a: A549 cells; Lane 8a: negative control. (**b**) A pooled DNA sample comprised equal amounts of members of the *P. brasiliensis* complex, *P. lutzii*, and DNA from A549 cells, a cell line derived from a human pulmonary adenocarcinoma. The absence of cross-reactions with human DNA was confirmed by removing *Paracoccidioides* DNA from the pooled sample. Lane 1b: Pb18 (S1), Pb01 (*P. lutzii*), and A549 cells; Lane 2b: EPM204 (PS2), Pb01, and A549 cells; Lane 3b: EPM77 (PS3), Pb01, and A549 cells; Lane 4b: EPM73 (PS4), Pb01, and A549 cells; Lane 5b: Pb18, EPM204, EPM77, EPM73, and A549 cells; Lane 6b: Pb01 and A549 cells; Lane 7b: A549 cells; Lane 8B: negative control. Data are representative of two independent experiments.

The specificity of amplification and the ability to detect mixed infection were evaluated using two different DNA templates in a duplex assay. We prepared a pool of any two *Paracoccidioides* DNA (50 ng/species), and in each case, fragments of 308 bp and 142 bp were resolved after gel electrophoresis, confirming the simultaneous presence of *P. brasiliensis* complex and *P. lutzii* DNA in the pool. When one of the target DNA was excluded from the PCR, a single amplicon was detected (308 bp or 142 bp), supporting the potential use of this test to detect mixed samples (Figure 4a). Thereafter, DNA from A549 cells was spiked with equal amounts of *Paracoccidioides* DNA and used to confirm that our assay did not amplify human DNA. Reactions containing A549 derived DNA spiked with *Paracoccidioides* species were positive, whereas non-spiked A549 samples remained negative after PCR (Figure 4b), supporting our in silico findings using BLASTn-searches [44]. Therefore, our test did not show any cross-reaction with human DNA that could possibly lead to misinterpretation of the presence/absence of target fungal DNA in a clinical sample.

A single round of PCR was used to evaluate the analytical sensitivity of each primer pair by using 10-fold serial DNA dilutions, starting at a concentration of 100 ng/μL. The limit of DNA detection was as little as 1 pg for both targets. The duplex PCR assay’s analytical sensitivities were equivalent to the test performed in a singleplex PCR format (i.e., 1 pg), showing that a duplex format did not affect the sensitivity of our assay.

**Figure 5:**
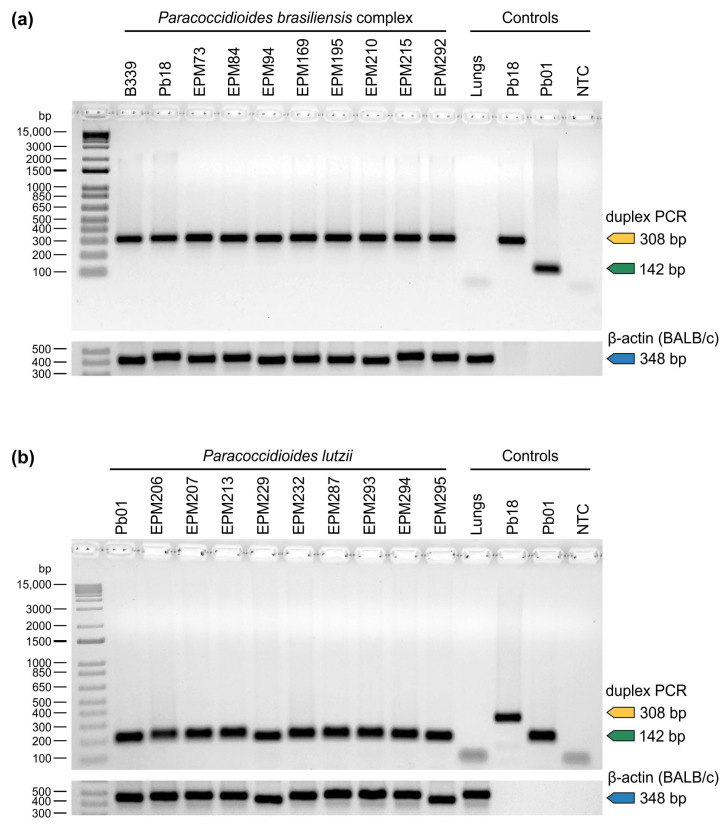
The potential use of our duplex PCR assay for detection studies. Agarose gel electrophoresis showing successful detection of *Paracoccidioides* DNA from spiked BALB/c lungs, using a duplex PCR assay. (**a**) BALB/c lungs spiked with *Paracoccidioides brasiliensis* yeasts (1 × 10^5^ yeasts). (**b**) BALB/c lungs spiked with *Paracoccidioides lutzii* yeasts (1 × 10^5^ yeasts). Controls include BALB/c lungs not spiked with *Paracoccidioides*; Pb18: *P. brasiliensis* complex positive control; Pb01: *P. lutzii* positive control; NTC: no template control. Lower panels represent the DNA quality control for BALB/c assessed by amplifying the β-actin gene. A positive β-actin amplification (348 bp) confirmed that the samples were free of PCR inhibitors. Data are representative of two independent experiments.

We evaluated if our duplex PCR assay could selectively detect *Paracoccidioides* DNA from soil and host samples. We successfully detected positive amplification from the lungs of mice spiked with *Paracoccidioides* spp. yeasts (Figure 5), indicating the potential use of fresh tissue fragments to detect *Paracoccidioides*. A 10-fold dilution was used to assess our assay’s ability to detect *Paracoccidioides* DNA in the presence of soil DNA. Therefore, the limit of *Paracoccidioides* DNA detection in soil with UV-transillumination was as little as 1 pg for both targets. Moreover, no false positives were detected in samples from the control group (non-spiked samples). Thus, it can be assumed that our assay can selectively detect pathogen DNA, confirming the high specificity and its potential applicability to detection studies.

**Figure 6:**
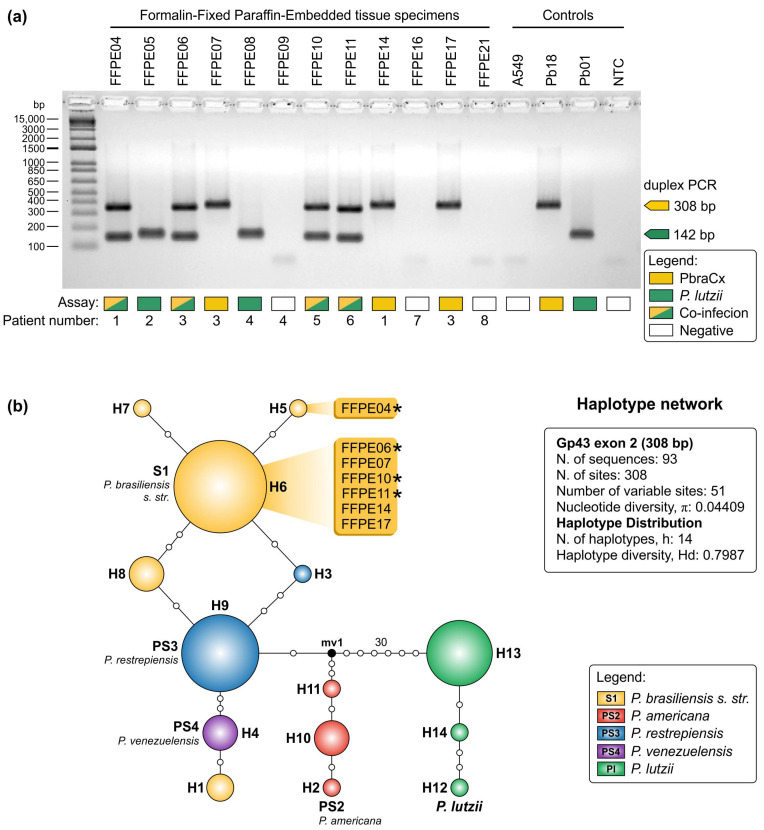
Detection of *Paracoccidioides* DNA from formalin-fixed, paraffin-embedded (FFPE) tissue sections. (**a**) Agarose gel electrophoresis showing successful detection of *Paracoccidioides* DNA using a duplex PCR assay. Remarkably, in four cases (i.e., FFPE04, FFPE06, FFPE10, and FFPE11), we confirmed co-infection due to *P. brasiliensis s.l.* and *P. lutzii*. Controls include DNA from A549 cells; Pb18: *P. brasiliensis* complex positive control; Pb01: *P. lutzii* positive control; NTC: no template control. (**b**) Median-joining haplotype network of *Paracoccidioides* species, covering all the 308-bp FFPE-derived sequences found in this study. The circumference diameter is proportional to the frequency of the haplotype. White dots represent mutational steps, and asterisks correspond to co-infections. Unsampled or extinct haplotypes in the population are indicated as black dots (median vectors). FFPE-derived sequences were clustered in two haplotypes (H5 or H6). Further information about sequence sources and GenBank accession numbers can be found in Supplementary Table S2.

DNA was successfully extracted from 12 FFPE biopsies from patients with a histological diagnosis consistent with paracoccidioidomycosis. *Paracoccidioides* DNA was detected in sections of tibia, duodenum, lungs, lymph node, oral mucosa, and skin (Supplementary Table S3). Attempts at amplicon sequencing produced good quality sequences, confirming our assay’s specificity (Accession numbers: MW556435-MW556441). Altogether, we were successful for 75% (9 out 12) of the FFPE samples tested in detecting *Paracoccidioides* DNA due to members of the *P. brasiliensis* complex (*n* = 7) or *P. lutzii* (*n* = 6) and in four cases (i.e., FFPE04, FFPE06, FFPE10, and FFPE11), we confirmed co-infection due to *P. brasiliensis s.l.* and *P. lutzii* at the same block by gel electrophoresis (Figure 6a).

Haplotype analysis included sequences generated in this study and reference sequences from well-characterized strains (*n* = 93; Supplementary Table S2). The final dataset had 308 characters, of which 51 were variable, 47 parsimony-informative, and 4 singletons. Genetic variability expressed by haplotype (Hd = 0.7987) and nucleotide (π = 0.04409) diversities were high in our dataset, and the sequences were spread into 14 haplotypes covering *P. brasiliensis s. str.* (h = 5), *P. americana* (h = 3), *P. restrepiensis* (h = 2), *P. venezuelensis* (h = 1), and *P. lutzii* (h = 3). Most haplotypes belonged to the S1 group and six of the gp43 FFPE-derived sequences (FFPE06_Pb, FFPE07_Pb, FFPE10_Pb, FFPE11_Pb, FFPE14_Pb, and FFPE17_Pb) clustered in H6. The exception was FFPE04_Pb, which presented a nonsynonymous transition (C→T) at position 200, leading to a switch from a leucine (L) to phenylalanine (F), and was classified in haplotype 5 (H5). Although gel electrophoresis could not differentiate among the *P. brasiliensis* complexes, DNA sequencing and haplotype network analysis supports the identification of *P. brasiliensis s. str.* (Figure 6b). Interestingly, patient #1 was diagnosed with a co-infection due to *P. lutzii* (FFPE04) and two genotypes of *P. brasiliensis s. str.* (FFPE04 = H5 and FFPE14 = H6), demonstrating the potential of our duplex PCR assay when associated with DNA sequencing analysis.

Sequences generated for *P. lutzii* amplicons were not included in the haplotype analysis, as they were considerably shorter (142 bp), and no polymorphisms were detected in the *P. lutzii* FFPE-derived sequences. A BLAST-search revealed 100% similarity (e-value: 2 × 10^−66^) of our six sequences (i.e., FFPE04_Pl, FFPE05_Pl, FFPE06_Pl, FFPE08_Pl, FFPE10_Pl, and FFPE11_Pl) with the isolate Pb01 (Accession number: XM_002792442.2; Supplementary Table S3).

## 4. Discussion

In this study, a duplex PCR assay was developed to simultaneously detect and differentiate between members of the *P. brasiliensis* complex and *P. lutzii*. Our assay has the following advantages: (i) high specificity and sensitivity for *Paracoccidioides* species; (ii) amplification of two targets alongside in a single reaction round, decreasing the amount of PCR reagents and number of steps required to identify *Paracoccidioides* species; (iii) higher sensitivities than other tests using conventional PCR; (iv) no amplification of human DNA, supporting use directly from clinical samples such as FFPE; (v) low cost compared to other molecular methods such as DNA-sequencing or qPCR methods; (vi) quick results (short processing time); and (vii) straightforward interpretation based on detection with UV-transillumination.

Diagnosis of paracoccidioidomycosis relies mainly on a combination of radiological, clinical, and laboratory findings [34,60,61]. From a clinical point of view, to date, no significant differences have been reported in the clinical signs of paracoccidioidomycosis caused by members of the *P. brasiliensis* complex (e.g., *P. brasiliensis s. str.* and *P. americana*) [28] or even between *P. brasiliensis sensu lato* and *P. lutzii* [5,29,62]. Most cases are classified as being of a chronic form, and lungs and lymph nodes are the most affected organs [5,28,29]. The reference methods for PCM diagnosis include direct examination of sputum and other secretions of patients and tissue sections or the isolation of *Paracoccidioides* in vitro. Positive samples reveal pathognomonic birefringent and multi-budding yeasts. However, different *Paracoccidioides* species show overlapping phenotypes, especially among closely related genetic clusters [24], which does not differentiate down to the group level (S1, PS2, PS3, PS4, and *P. lutzii*) using morphology alone [34]. On the other hand, serological assays aimed at detecting circulating antibodies such as the double immunodiffusion or ELISA highlight the main difference related to *P. brasiliensis s.l.* and *P. lutzii* diagnosis, but there is a need to employ local antigenic preparations in serodiagnosis to avoid false-negative results [63,64]. Therefore, our test can be an essential tool in these paradoxical scenarios, where positive results are found in direct examination of biological fluids or histopathology, but negative results are found using serological assays [5,63,65,66,67,68,69].

Another strength of our study is the possibility of detecting two species in a single reaction (e.g., any member of the *P. brasiliensis* complex along with *P. lutzii*), demonstrating, for example, mixed cultures in primary isolation or putative co-infection directly from clinical samples, opening new opportunities for diagnosis, and the main factors potentially implicated in the etiopathogenesis of PCM [70]. This is supported by the assay’s excellent sensitivity, which allowed us to detect positive results using as little as 1 pg of *Paracoccidioides* DNA, along with the absence of amplification of human DNA using a cell line derived from pulmonary adenocarcinoma or in the murine samples. Our duplex assay goes further, since we demonstrated the high specificity (100%) by testing 62 *Paracoccidioides* samples in addition to 68 isolates representing non-target DNA, with no false-positive or false-negative results.

As a proof of concept, a landmark of our study is the description, for the first time, of mixed infections in humans consisting of both *P. brasiliensis s. str.* and *P. lutzii* detected in a wide range of PCM patient’s organs living in endemic areas in the São Paulo state, Brazil. The molecular strategy used in our assay to investigate FFPE tissues allows the differentiation of *P. brasiliensis* complex and *P. lutzii*. Notwithstanding, we highlight that it is not possible to discriminate members of the *P. brasiliensis* complex (S1, PS2, PS3, and PS4) by gel electrophoresis, as the amplicons have the same size (i.e., 308 bp), thus supporting the use of DNA sequencing for identification down to species level. From an epidemiological perspective, our findings are relevant, as it allows us to recognize and estimate the frequency of mixed infections in South America countries, a point that had never been investigated before in the history of this centenarian mycosis. Although the co-infections reported here were predominantly due to *P. brasiliensis s. str.* (S1) and *P. lutzii*, the incidence of any member of the *P. brasiliensis* complex and *P. lutzii* may occur. From a laboratorial perspective, the isolation and characterization of *Paracoccidioides* isolated from patients remain the effective method to demonstrate mixed infections. However, the use of non-selective media most likely favors the isolation of a single genotype in *Paracoccidioides*. The direct identification of simultaneous infections using PCR/sequencing applied to FFPE may present a considerable advantage over culture typing due to the high sensitivity of the PCR [54,71].

From a clinical perspective, PCM progression varies significantly from host to host, and etiopathology may be affected by several factors such as age, sex, immunity status, and genetics. In this scenario, mixed infections may impact treatment and outcome. Great examples show that co-infections and reinfections can exacerbate disease progression, subsequently affecting patients’ clinical management [72]. For example, mixed *Plasmodium* malaria infections can lead to severe malaria [72]. Reinfection and mixed *Trypanosoma cruzi* infections impact Chagas disease progression [71], and human mixed infections of *Leishmania* spp. and *Leishmania*-*Trypanosoma cruzi* have consequences to the diseases’ control and patient treatments [73].

Considering that mixed fungal infections exist and that more than one species could be involved, the diagnosis should not rely on identifying a single colony and a unique sampling site [74,75,76]. Guarro et al. identified a strain of *Fusarium verticillioides* from blood culture and *F. solani* from a skin biopsy, both from the same HIV-positive patient [77]. The DNA extracted from FFPE samples allowed Ricci et al. to describe the molecular epidemiology of a cluster of *Pneumocystis jirovecii* pneumonia in renal transplant recipients, uncovering that 4 out 11 patients were infected by more than one genotype [78]. Ponzio et al. have identified patients infected with various molecular siblings of *Cryptococcus neoformans*, such as VNI and VNII, isolated from infected patients’ cerebrospinal fluid or blood [79]. Therefore, detection of only one species when more species are present may result in erroneous treatment [74,75,76,77,79,80]. Likewise, we argue that mixed *Paracoccidioides* spp. infections could determine the primary outcome of the patient. Further studies should be performed with a substantial number of clinical samples and a clinical survey to confirm or refute this hypothesis.

The high genetic diversity is remarkable in *Paracoccidioides*, which leads to the description of several cryptic species [14,81]. Restriction fragment length polymorphism (RFLP) first figured in the PCM in studies performed by Niño-Vega et al. [10] to identify the tremendous genetic diversity and band patterns using *Hinf*I or *Hinc*II restriction enzymes, which grouped the isolates according to the geographical origin. However, this is not adequate for taxonomic purposes, since it cannot recognize all cryptic groups in *Paracoccidioides* [81]. Randomly amplified polymorphic DNA (RAPD) has shown strong discriminating power, separating isolates geographically, but limitations include low experimental reproducibility, difficult interpretation of results, and failure to speciate *Paracoccidioides* [10,81].

The first attempts at genotyping *Paracoccidioides* isolates were based on the sequence polymorphism of nuclear and mitochondrial markers, which were responsible for introducing cryptic species to the *P. brasiliensis* complex, leading to the proposal of new phylogenetic groups and a novel species, *P. lutzii* [17,20,31]. Multilocus sequence analysis is, therefore, considered the reference method to recognize these taxonomic entities and has been supported by studies involving whole-genome sequencing followed by phylogenomic analysis of *Paracoccidioides* species [82].

After speciation in *Paracoccidioides*, few methods have been described aiming at the detection and/or rapid identification of isolates. A PCR-based assay targeting an indel region in the HSP70 locus can reportedly selectively amplify *P. lutzii* isolates [19], but negative results do not indicate the presence of *P. brasiliensis*, a limitation of the assay. Our duplex assay, on the other hand, can help overcome this drawback. A PCR-RFLP was proposed by Roberto et al. [32], enabling a new approach to recognize cryptic species in *Paracoccidioides*. Although fairly accurate, the test requires a previously well-characterized strain from each of the cryptic species to be used as a positive control during electrophoresis. Moreover, the *TUB1*-RFLP was designed before the PS4 description (*P. venezuelensis*) and may not cover all species in the *P. brasiliensis* complex [34]. Here, all cryptic species in the *P. brasiliensis* complex were covered and presented positive results in amplification with PbraCx-F and PbraCx-R primers.

A total of five microsatellite markers were used to differentiate members of the *P. brasiliensis* complex, including S1, PS2, and PS3 groups. The two major groups (S1 and PS2) could be distinguished by microsatellite typing, but this method has comparatively low discriminatory power to recognize PS3 genotypes, mostly due to the low intraspecific diversity [13,17,24]. Recently, Alves et al. [83] explored the transposable elements of Trem A-H’s potential use as a typing method for *Paracoccidioides* species. The test took advantage of the high copy numbers and distribution of these mobile elements in the *Paracoccidioides* genomes. However, like microsatellite markers [13], Trem elements failed to discriminate cryptic species in the *P. brasiliensis* complex [83].

Theodoro et al. [24] proposed to detect single nucleotide polymorphisms using SNaPshot and/or qPCR to recognize *Paracoccidioides* species. SNaPshot requires two PCR amplifications (i.e., arf and gp43) followed by enzymatic treatments and capillary electrophoresis, increasing the diagnosis price. Likewise, the qPCR assay proposed by [24] requires three primer pairs targeting arf, gp43, and PRP8 and six probes targeting SNPs in *Paracoccidioides* species using three reactions, increasing the complexity and price of the test to characterize an isolate fully.

The main clinical sample employed in routine diagnosis is sputum and tissue biopsies, including paraffin-embedded tissues [34]. Protocols using conventional PCR showed good performance on sputum but not in serum samples, with the lower limit of detection being 10 cell/mL and 1.1 pg, respectively [33,84]. Here, we demonstrate that our assay performs well in the presence of human and murine DNA, with sensitivities comparable for the detection of the fungus on sputum (1 fg) [85] or using tissue biopsies (0.25 pg) [84]. The merit of our protocol is its simplicity, low cost, high sensitivity and high specificity, and suitability for multiple purposes, including, for the first time, detection of co-infection by any member of the *P. brasiliensis* complex and *P. lutzii*, without the need for DNA sequencing. This is a significant advantage since many tests presented previously do not consider the detection of *P. lutzii*. There is, however, a need for validation with a substantial number of clinical samples. Nevertheless, these results encourage the detection of *Paracoccidioides* in clinical routine and highlight that obtaining an excellent clinical specimen is the first significant step in maintaining diagnostic accuracy [34].

## 5. Conclusions

There are still only a limited number of DNA-based fingerprinting methods available for typing newly described species, most of them using a low number of samples, and these methods may not all be suitable in cases of high intraspecific genetic variability, producing false positive or false negative results, assigning isolates to taxa divergent from the classification using multilocus sequence analysis or whole-genomic sequencing. Therefore, for diagnostic purposes, the strategy used here to differentiate *P. brasiliensis* complex and *P. lutzii* facilitates the interpretation of the results and minimizes the errors presented by other tests that generate dubious characterizations, require multiple steps, and/or are expensive for the diagnosis of a neglected disease occurring mainly in upper–middle-income countries.

## Figures and Tables

**Table 1:** Species-specific primer sequences that targeted the gp43 gene (exon 2) in members of the genus *Paracoccidioides*.

Target Species	Primer	Primer Sequence (5’-3’)	Amplicon Size
*P. brasiliensis* complex	PbraCx-F	TCG TGA TAT AGA CAG CAC CGT TG	308 bp
	PbraCx-R	ACG AAC CAT CAA ATC GCG AAC CTA	
*P. lutzii*	Plu-F	CTT CAT GGC GCC CAA GGA CTT	142 bp
	Plu-R	GAC TCC GCT AAG TCT GTC CTT AGG C	

## Data Availability

The data presented in this study are available within the article and supplementary material. Sequences are available at GenBank (https://www.ncbi.nlm.nih.gov/genbank/) under the accession numbers MW556431–MW556441.

## Supplementary Materials

The following are available online at https://www.mdpi.com/2309-608X/7/3/169/s1, Table S1: *Paracoccidioides* isolates used in this study. Table S2. Sequences available at Genbank, used for primer design. Table S3. List of formalin-fixed paraffin-embedded (FFPE) tissue samples included in the analysis. Table S4. Primer-BLAST results. Target templates were found in the selected database: Nucleotide collection (nt) (Organism limited to Fungi, as the other taxa searches did not return amplicons).
