# Supplementary material for: A New Duplex PCR-Assay for the Detection and Identification of Paracoccidioides Species
Source: J Fungi (Basel). 2021 Feb 26;7(3):169. doi: 10.3390/jof7030169 (PMC7996757; doi:10.3390/jof7030169)
Supplement: Supplementary file 1 [file jof-07-00169-s001.pdf]

Journal of *Fungi*

MDPI

Supplementary material

Supplementary material: A new duplex PCR-assay for detection and identification of *Paracoccidioides* species

Breno Gonçalves Pinheiro[1], Ana Paula Pôssa[1,2], Paula Portella Della Terra[1,2], Jamile Ambrósio de Carvalho[1], Giannina Ricci[3], Angela Satie Nishikaku[3], Rosane Christine Hahn[4,5], Zoilo Pires de Camargo[1,2], Anderson Messias Rodrigues[1,2*]

[1] Laboratory of Emerging Fungal Pathogens, Department of Microbiology, Immunology, and Parasitology, Discipline of Cellular Biology, Federal University of São Paulo (UNIFESP), São Paulo 04023062, Brazil.
[2] Department of Medicine, Discipline of infectious Diseases, Federal University of São Paulo (UNIFESP), São Paulo 04023062, Brazil.
[3] Centro de Diagnóstico e Pesquisa em Biologia Molecular Dr. Ivo Ricci, São Carlos 13561020, São Paulo, Brazil.
[4] Laboratory of Mycology/Research, Faculty of Medicine, Federal University of Mato Grosso, Cuiabá 78060900, Brazil.
[5] Júlio Muller University Hospital, Federal University of Mato Grosso, Cuiabá 78048902, Brazil.
* Correspondence: amrodrigues.amr@gmail.com; Tel.: +55-1155764551 Ext. 1540.

Clinical and environmental *Paracoccidioides* spp. strains described in Table S1 were obtained from the culture collection of Federal University of São Paulo (UNIFESP), São Paulo, Brazil. Isolates were kept as slants on Fava-Netto medium at 37 °C in yeast form. These isolates were previously characterized down to species level by *TUB1*-RFLP as previously described by Roberto *et al.* [1]. Reference strains (e.g., Pb18, Pb03, B339, Pb01) representing the main *Paracoccidioides* species covering a plethora of genotypes were included in all experiments.

Table S1. *Paracoccidioides* isolates used in this study.

Isolate	Other code	*TUB1*-RFLP	Duplex PCR	Reference
EPM01	B339	PS3	*P. brasiliensis* complex	[1]
EPM09	2GB-ES	S1	*P. brasiliensis* complex	[1]
EPM10	Bt03	S1	*P. brasiliensis* complex	[1]
EPM16	Pb18	S1	*P. brasiliensis* complex	[1]
EPM18	Colombo	S1	*P. brasiliensis* complex	[1]
EPM30	IBIA	PS3	*P. brasiliensis* complex	[1]
EPM32	38550Niterói	S1	*P. brasiliensis* complex	[1]
EPM33	1789.88	S1	*P. brasiliensis* complex	[1]
EPM35	1965Uruguai	PS2	*P. brasiliensis* complex	[1]
EPM38	15959	S1	*P. brasiliensis* complex	[1]
EPM46	CM	S1	*P. brasiliensis* complex	[1]
EPM56	350	S1	*P. brasiliensis* complex	[1]
EPM58	729	S1	*P. brasiliensis* complex	[1]
EPM61	2511	PS3	*P. brasiliensis* complex	[1]
EPM63	5736	S1	*P. brasiliensis* complex	[1]

Isolate	Other code	*TUB1*-RFLP	Duplex PCR	Reference
EPM73	9227	PS4	*P. brasiliensis* complex	[1]
EPM74	9570	PS4	*P. brasiliensis* complex	[1]
EPM77	JDA-80	PS3	*P. brasiliensis* complex	[1]
EPM78	Pb303	PS4	*P. brasiliensis* complex	[1]
EPM80	Pb308	S1	*P. brasiliensis* complex	[1]
EPM81	MSCol	PS3	*P. brasiliensis* complex	[1]
EPM84	Pb309	S1	*P. brasiliensis* complex	[1]
EPM85	32893	S1	*P. brasiliensis* complex	[1]
EPM87	Pb320	PS2	*P. brasiliensis* complex	[1]
EPM90	Pb324	PS2	*P. brasiliensis* complex	[1]
EPM108	20907	S1	*P. brasiliensis* complex	[1]
EPM112	Ibiá-T_3	S1	*P. brasiliensis* complex	[1]
EPM113	113	S1	*P. brasiliensis* complex	[1]
EPM118	D	S1	*P. brasiliensis* complex	[1]
EPM122	H	S1	*P. brasiliensis* complex	[1]
EPM127	24266	S1	*P. brasiliensis* complex	[1]
EPM135	Pb67	PS2	*P. brasiliensis* complex	[1]
EPM139	Pb75	S1	*P. brasiliensis* complex	[1]
EPM147	Pb01	*P. lutzii*	*P. lutzii*	[1]
EPM148	8941	*P. lutzii*	*P. lutzii*	[1]
EPM152	29791	S1	*P. brasiliensis* complex	[1]
EPM153	-	S1	*P. brasiliensis* complex	[1]
EPM165	Pb-MYC	S1	*P. brasiliensis* complex	[1]
EPM198	924	S1	*P. brasiliensis* complex	[1]
EPM204	1017	PS2	*P. brasiliensis* complex	[1]
EPM206	Pb66	*P. lutzii*	*P. lutzii*	[1]
EPM208	ED01	*P. lutzii*	*P. lutzii*	[1]
EPM213	Pb1744	*P. lutzii*	*P. lutzii*	[1]
EPM215	-	PS2	*P. brasiliensis* complex	[1]
EPM223	9840	*P. lutzii*	*P. lutzii*	[1]
EPM225	M-156	*P. lutzii*	*P. lutzii*	[1]
EPM232	3909	*P. lutzii*	*P. lutzii*	[1]
EPM239	4653	*P. lutzii*	*P. lutzii*	[1]
EPM242	4652	*P. lutzii*	*P. lutzii*	[1]
EPM249	LCF3116	S1	*P. brasiliensis* complex	[1]
EPM250	T19F18-1	S1	*P. brasiliensis* complex	[1]
EPM254	T19F24-1	S1	*P. brasiliensis* complex	[1]
EPM255	T19F33-1	S1	*P. brasiliensis* complex	[1]
EPM256	T23LM1-7	S1	*P. brasiliensis* complex	[1]
EPM258	T22LM1-1	S1	*P. brasiliensis* complex	[1]
EPM259	T19B14-1	S1	*P. brasiliensis* complex	[1]
EPM260	T22LM1-2	S1	*P. brasiliensis* complex	[1]

Isolate	Other code	*TUB1*-RFLP	Duplex PCR	Reference
EPM261	8652	S1	*P. brasiliensis* complex	[1]
EPM263	T23LM3-1	S1	*P. brasiliensis* complex	[1]
EPM264	T20B13-1	S1	*P. brasiliensis* complex	[1]
EPM271	5463	S1	*P. brasiliensis* complex	[1]
EPM273	BWMS	S1	*P. brasiliensis* complex	[1]
EPM276	Pb209/15	S1	*P. brasiliensis* complex	[1]
EPM283	5953	*P. lutzii*	*P. lutzii*	[1]
EPM284	5994	*P. lutzii*	*P. lutzii*	[1]
EPM285	6142	*P. lutzii*	*P. lutzii*	[1]
EPM287	6226	*P. lutzii*	*P. lutzii*	[1]
EPM288	6268	*P. lutzii*	*P. lutzii*	[1]
EPM289	6333	*P. lutzii*	*P. lutzii*	[1]
EPM290	HSP	S1	*P. brasiliensis* complex	[1]
EPM291	M696	S1	*P. brasiliensis* complex	[1]
EPM292	6414	S1	*P. brasiliensis* complex	[1]
EPM293	6454	*P. lutzii*	*P. lutzii*	[1]
EPM294	6455	*P. lutzii*	*P. lutzii*	[1]
EPM295	6507	*P. lutzii*	*P. lutzii*	[1]
EPM297	6445	*P. lutzii*	*P. lutzii*	[1]
EPM298	KLS	S1	*P. brasiliensis* complex	[1]

Journal of Fungi

Table S2. Sequences available at Genbank, used for primer design.

Isolate	Species	Genetic Group	Origin	Genbank	Reference
189	*P. lutzii*	*P. lutzii*	Brazil	EU870217	[2]
206	*P. lutzii*	*P. lutzii*	Brazil	EU870225	[2]
351	*P. lutzii*	*P. lutzii*	Brazil	EU870222	[2]
397	*P. lutzii*	*P. lutzii*	Brazil	EU870216	[2]
57	*P. lutzii*	*P. lutzii*	Brazil	EU870224	[2]
61	*P. lutzii*	*P. lutzii*	Brazil	EU870219	[2]
84	*P. lutzii*	*P. lutzii*	Brazil	EU870221	[2]
EPM212	*P. lutzii*	*P. lutzii*	Brazil	JX065607	[3]
EPM213	*P. lutzii*	*P. lutzii*	Brazil	JX065608	[3]
9840	*P. lutzii*	*P. lutzii*	Brazil	KC732777	[4]
Raj2	*P. lutzii*	*P. lutzii*	Brazil	EU870215	[2]
717	*P. lutzii*	*P. lutzii*	Brazil	EU870220	[2]
7455	*P. lutzii*	*P. lutzii*	Brazil	EU870213	[2]
6810	*P. lutzii*	*P. lutzii*	Brazil	EU870227	[2]
218	*P. lutzii*	*P. lutzii*	Brazil	EU870218	[2]
3171	*P. lutzii*	*P. lutzii*	Brazil	EU870214	[2]
Pb01	*P. lutzii*	*P. lutzii*	Brazil	EU870212	[2]
417	*P. lutzii*	*P. lutzii*	Brazil	MH367530	Unpublished
A8	*P. brasiliensis*	S1	Argentina	DQ003785	[5]
B1	*P. brasiliensis*	S1	Brazil	DQ003724	[5]
B14	*P. brasiliensis*	S1	Brazil	DQ003737	[5]
B19	*P. brasiliensis*	S1	Brazil	DQ003742	[5]
B2	*P. brasiliensis*	S1	Brazil	DQ003725	[5]
B20	*P. brasiliensis*	S1	Brazil	DQ003743	[5]
B3	*P. brasiliensis*	S1	Brazil	DQ003726	[5]
B4	*P. brasiliensis*	S1	Brazil	DQ003727	[5]
B5	*P. brasiliensis*	S1	Brazil	DQ003728	[5]
A2	*P. brasiliensis*	S1	Argentina	DQ003777	[5]
A3	*P. brasiliensis*	S1	Argentina	DQ003788	[5]
A5	*P. brasiliensis*	S1	Argentina	DQ003782	[5]
A7	*P. brasiliensis*	S1	Argentina	DQ003784	[5]
B10	*P. brasiliensis*	S1	Brazil	DQ003733	[5]
B11	*P. brasiliensis*	S1	Brazil	DQ003734	[5]
B12	*P. brasiliensis*	S1	Brazil	DQ003735	[5]
B16	*P. brasiliensis*	S1	Brazil	DQ003739	[5]
B17	*P. brasiliensis*	S1	Brazil	DQ003740	[5]
B22	*P. brasiliensis*	S1	Brazil	DQ003745	[5]
B24	*P. brasiliensis*	S1	Brazil	DQ003747	[5]
B25	*P. brasiliensis*	S1	Brazil	DQ003748	[5]
B6	*P. brasiliensis*	S1	Brazil	DQ003729	[5]
B9	*P. brasiliensis*	S1	Brazil	DQ003732	[5]
U1	*P. brasiliensis*	S1	Uruguay	DQ003778	[5]
A4	*P. brasiliensis*	S1	Argentina	DQ003781	[5]
B8	*P. brasiliensis*	S1	Brazil	DQ003731	[5]
A1	*P. brasiliensis*	S1	Argentina	DQ003780	[5]
A6	*P. brasiliensis*	S1	Argentina	DQ003783	[5]
B21	*P. brasiliensis*	S1	Brazil	DQ003744	[5]

Isolate	Species	Genetic Group	Origin	Genbank	Reference
P1	*P. brasiliensis*	S1	Paraguay	DQ003786	[5]
P2	*P. brasiliensis*	S1	Paraguay	DQ003787	[5]
B18	*P. brasiliensis*	S1	Brazil	DQ003741	[5]
C1	*P. brasiliensis*	PS3	Colombia	DQ003750	[5]
C10	*P. brasiliensis*	PS3	Colombia	DQ003759	[5]
C11	*P. brasiliensis*	PS3	Colombia	DQ003760	[5]
C12	*P. brasiliensis*	PS3	Colombia	DQ003761	[5]
C13	*P. brasiliensis*	PS3	Colombia	DQ003762	[5]
C14	*P. brasiliensis*	PS3	Colombia	DQ003763	[5]
C15	*P. brasiliensis*	PS3	Colombia	DQ003764	[5]
C16	*P. brasiliensis*	PS3	Colombia	DQ003765	[5]
C17	*P. brasiliensis*	PS3	Colombia	DQ003766	[5]
C18	*P. brasiliensis*	PS3	Colombia	DQ003767	[5]
C19	*P. brasiliensis*	PS3	Colombia	DQ003768	[5]
C2	*P. brasiliensis*	PS3	Colombia	DQ003751	[5]
C20	*P. brasiliensis*	PS3	Colombia	DQ003769	[5]
C21	*P. brasiliensis*	PS3	Colombia	DQ003770	[5]
C3	*P. brasiliensis*	PS3	Colombia	DQ003752	[5]
C4	*P. brasiliensis*	PS3	Colombia	DQ003753	[5]
C7	*P. brasiliensis*	PS3	Colombia	DQ003756	[5]
C5	*P. brasiliensis*	PS3	Colombia	DQ003754	[5]
C6	*P. brasiliensis*	PS3	Colombia	DQ003755	[5]
C8	*P. brasiliensis*	PS3	Colombia	DQ003757	[5]
C9	*P. brasiliensis*	PS3	Colombia	DQ003758	[5]
V1	*P. brasiliensis*	PS4	Venezuela	DQ003771	[5]
V3	*P. brasiliensis*	PS4	Venezuela	DQ003773	[5]
V5	*P. brasiliensis*	PS4	Venezuela	DQ003775	[5]
V4	*P. brasiliensis*	PS4	Venezuela	DQ003774	[5]
V6	*P. brasiliensis*	PS4	Venezuela	DQ003776	[5]
B13	*P. brasiliensis*	PS2	Brazil	DQ003736	[5]
B15	*P. brasiliensis*	PS2	Brazil	DQ003738	[5]
V2	*P. brasiliensis*	PS2	Venezuela	DQ003772	[5]
B23	*P. brasiliensis*	PS2	Brazil	DQ003746	[5]
B7	*P. brasiliensis*	PS2	Brazil	DQ003730	[5]
B26	*P. brasiliensis*	PS2	Brazil	DQ003749	[5]

Table S3. List of formalin-fixed paraffin-embedded (FFPE) tissue samples included in the analysis.

Code	Patient	Year	Organ	Gender	Age	Origin[1]	Genbank *P. bra* cx	Genbank *P. lutzii* [2]
FFPE04	1	2011	Tibia	Male	18	São Paulo, SP, Brazil	**MW556435**	**Positive**
FFPE05	2	2010	Lymph node	Male	42	Juiz de Fora, MG, Brazil	Negative	**Positive**
FFPE06	3	2011	Lymph node	Male	13	São Paulo, SP, Brazil	**MW556436**	**Positive**
FFPE07	3	2011	Lymph node	Male	13	São Paulo, SP, Brazil	**MW556437**	Negative
FFPE08	4	2019	Oral mucosa	Male	50	São Carlos, SP, Brazil	Negative	**Positive**
FFPE09	4	2019	Oral mucosa	Male	50	São Carlos, SP, Brazil	Negative	Negative
FFPE10	5	2019	Skin	Male	58	São Carlos, SP, Brazil	**MW556438**	**Positive**
FFPE11	6	2014	Duodenum	Female	35	São Carlos, SP, Brazil	**MW556439**	**Positive**
FFPE14	1	2011	Tibia	Male	18	São Paulo, SP, Brazil	**MW556440**	Negative
FFPE16	7	2016	Lungs	Male	63	São Paulo, SP, Brazil	Negative	Negative
FFPE17	3	2011	Lymph node	Male	13	São Paulo, SP, Brazil	**MW556441**	Negative
FFPE21	8	2020	Skin	Male	30	São Carlos, SP, Brazil	Negative	Negative

[1]SP: São Paulo; MG: Minas Gerais. [2]Sequences <200 bp long are not accepted by GenBank, therefore, positive *P. lutzii* sequences are listed below:

>FFPE04
CTTCATGGCGCCCAAGGACTTCAAGAACGTCCACCTCGACACACACCACTACCAAGTCTTCGATGATGCCTTTAAGA
CCTTCACCATCGACCAGCACGTGAAGCTTGCATGCTCGCTGCCTAAGGACAGACTTAGCGGAGTC

>FFPE05
CTTCATGGCGCCCAAGGACTTCAAGAACGTCCACCTCGACACACACCACTACCAAGTCTTCGATGATGCCTTTAAGA
CCTTCACCATCGACCAGCACGTGAAGCTTGCATGCTCGCTGCCTAAGGACAGACTTAGCGGAGTC

>FFPE06
CTTCATGGCGCCCAAGGACTTCAAGAACGTCCACCTCGACACACACCACTACCAAGTCTTCGATGATGCCTTTAAGA
CCTTCACCATCGACCAGCACGTGAAGCTTGCATGCTCGCTGCCTAAGGACAGACTTAGCGGAGTC

>FFPE08
CTTCATGGCGCCCAAGGACTTCAAGAACGTCCACCTCGACACACACCACTACCAAGTCTTCGATGATGCCTTTAAGA
CCTTCACCATCGACCAGCACGTGAAGCTTGCATGCTCGCTGCCTAAGGACAGACTTAGCGGAGTC

>FFPE10
CTTCATGGCGCCCAAGGACTTCAAGAACGTCCACCTCGACACACACCACTACCAAGTCTTCGATGATGCCTTTAAGA
CCTTCACCATCGACCAGCACGTGAAGCTTGCATGCTCGCTGCCTAAGGACAGACTTAGCGGAGTC

>FFPE11
CTTCATGGCGCCCAAGGACTTCAAGAACGTCCACCTCGACACACACCACTACCAAGTCTTCGATGATGCCTTTAAGA
CCTTCACCATCGACCAGCACGTGAAGCTTGCATGCTCGCTGCCTAAGGACAGACTTAGCGGAGTC

Journal of Fungi

MDPI

Table S4. Primer-BLAST results. Target templates were found in the selected database: Nucleotide collection (nt) (Organism limited to Fungi, as the other taxa searches did not return amplicons).

Search parameter name	Search parameter value
Number of Blast hits analyzed	11065
Entrez query	
Min total mismatches	2
Min 3' end mismatches	2
Defined 3' end region length	5
Mismatch threshold to ignore targets	6
Max target size	4000
Max number of Blast target sequences	50000
Blast E value	30000
Blast word size	7
Max candidate primer pairs	500
Min PCR product size	67
Max PCR product size	1000
Min Primer size	15
Opt Primer size	20
Max Primer size	25
Min Tm	57
Opt Tm	60
Max Tm	63
Max Tm difference	3
Repeat filter	AUTO
Low complexity filter	Yes

Primer pair 1: *Paracoccidioides brasiliensis* complex

	Sequence (5'->3')	Length	Tm	GC%	Self complementarity	Self 3' complementarity
**Forward primer**	TCGTGATATAGACAGCACCGTTG	23	60.49	47.83	4.00	4.00
**Reverse primer**	ACGAACCATCAAATCGCGAACCTA	24	62.93	45.83	6.00	2.00

Products on target templates

>MK909798.1 *Paracoccidioides brasiliensis* isolate HCRPFOZ_1 immunodominant antigen Gp43 (GP43) gene, partial cds

```
product length = 308

Forward primer  1    TCGTGATATAGACAGCACCGTTG  23

Template        60   .......................  82

Reverse primer  1    ACGAACCATCAAATCGCGAACCTA  24

Template        367  ........................  344
```

>MK909794.1 *Paracoccidioides brasiliensis* isolate HCRP193 immunodominant antigen Gp43 (GP43) gene, partial cds

```
product length = 308

Forward primer  1   TCGTGATATAGACAGCACCGTTG  23

Template        60  .......................  82

Reverse primer  1    ACGAACCATCAAATCGCGAACCTA  24

Template        367  ........................  344
```

>MH909768.1 *Paracoccidioides brasiliensis* isolate HCRP055 immunodominant antigen Gp43 (GP43) gene, partial cds

```
product length = 308

Forward primer  1   TCGTGATATAGACAGCACCGTTG  23

Template        60  .......................  82

Reverse primer  1    ACGAACCATCAAATCGCGAACCTA  24

Template        367  ........................  344
```

>MH484614.1 *Paracoccidioides brasiliensis* isolate BAT immunodominant antigen Gp43 (GP43) gene, partial cds

```
product length = 308

Forward primer  1   TCGTGATATAGACAGCACCGTTG  23

Template        23  .......................  45

Reverse primer  1    ACGAACCATCAAATCGCGAACCTA  24

Template        330  ........................  307
```

>MH367531.1 *Paracoccidioides brasiliensis* strain 531 immunodominant antigen Gp43 (GP43) gene, partial cds

```
product length = 308

Forward primer  1    TCGTGATATAGACAGCACCGTTG  23

Template        246  .......................  268

Reverse primer  1    ACGAACCATCAAATCGCGAACCTA  24

Template        553  ........................  530
```

>MH367530.1 *Paracoccidioides brasiliensis* strain 417 immunodominant antigen Gp43 (GP43) gene, partial cds

```
product length = 308

Forward primer  1    TCGTGATATAGACAGCACCGTTG  23

Template        246  .......................  268

Reverse primer  1    ACGAACCATCAAATCGCGAACCTA  24

Template        553  ........................  530
```

>MH367529.1 *Paracoccidioides brasiliensis* strain 326 immunodominant antigen Gp43 (GP43) gene, partial cds

```
product length = 308

Forward primer  1    TCGTGATATAGACAGCACCGTTG  23

Template        246  .......................  268

Reverse primer  1    ACGAACCATCAAATCGCGAACCTA  24

Template        553  ........................  530
```

>MH367528.1 *Paracoccidioides brasiliensis* strain 234 immunodominant antigen Gp43 (GP43) gene, partial cds

```
product length = 308

Forward primer  1    TCGTGATATAGACAGCACCGTTG  23

Template        246  .......................  268

Reverse primer  1    ACGAACCATCAAATCGCGAACCTA  24

Template        553  ........................  530
```

>MH367527.1 *Paracoccidioides brasiliensis* strain 192 immunodominant antigen Gp43 (GP43) gene, partial cds

```
product length = 308

Forward primer  1    TCGTGATATAGACAGCACCGTTG  23

Template        246  .......................  268

Reverse primer  1    ACGAACCATCAAATCGCGAACCTA  24

Template        553  ........................  530
```

>MH029222.1 *Paracoccidioides brasiliensis* strain 29572 immunodominant antigen Gp43 (gp43) gene, partial cds

```
product length = 308

Forward primer  1   TCGTGATATAGACAGCACCGTTG  23

Template        20  .......................  42

Reverse primer  1    ACGAACCATCAAATCGCGAACCTA  24

Template        327  ........................  304
```

>MH029217.1 *Paracoccidioides brasiliensis* strain 47976 immunodominant antigen Gp43 (gp43) gene, partial cds

```
product length = 308

Forward primer  1  TCGTGATATAGACAGCACCGTTG  23

Template        1  .......................  23

Reverse primer  1    ACGAACCATCAAATCGCGAACCTA  24

Template        308  ........................  285
```

>MH029209.1 *Paracoccidioides* sp. 'americana' strain 28434 immunodominant antigen Gp43 (gp43) gene, partial cds

```
product length = 308

Forward primer  1   TCGTGATATAGACAGCACCGTTG  23

Template        20  .......................  42

Reverse primer  1    ACGAACCATCAAATCGCGAACCTA  24

Template        327  ........................  304
```

>MF066649.1 *Paracoccidioides brasiliensis* strain IPEC 2019H immunodominant antigen gp43 gene, partial cds

```
product length = 308

Forward primer  1   TCGTGATATAGACAGCACCGTTG  23

Template        59  .......................  81

Reverse primer  1    ACGAACCATCAAATCGCGAACCTA  24

Template        366  ........................  343
```

>KY963817.1 *Paracoccidioides brasiliensis* isolate PSM immunodominant antigen Gp43 (GP43) gene, exon 2 and partial cds

```
product length = 308

Forward primer  1   TCGTGATATAGACAGCACCGTTG  23

Template        60  .......................  82

Reverse primer  1    ACGAACCATCAAATCGCGAACCTA  24

Template        367  ........................  344
```

>KY963816.1 *Paracoccidioides brasiliensis* isolate PbD immunodominant antigen Gp43 (GP43) gene, exon 2 and partial cds

```
product length = 308

Forward primer  1   TCGTGATATAGACAGCACCGTTG  23

Template        60  .......................  82

Reverse primer  1    ACGAACCATCAAATCGCGAACCTA  24

Template        367  ........................  344
```

>KY963820.1 *Paracoccidioides brasiliensis* isolate BACR immunodominant antigen Gp43 (GP43) gene, exon 2 and partial cds

```
product length = 308

Forward primer  1   TCGTGATATAGACAGCACCGTTG  23

Template        60  .......................  82

Reverse primer  1    ACGAACCATCAAATCGCGAACCTA  24

Template        367  ........................  344
```

>KY963819.1 *Paracoccidioides brasiliensis* isolate T23LM3-4 immunodominant antigen Gp43 (GP43) gene, exon 2 and partial cds

```
product length = 308

Forward primer  1   TCGTGATATAGACAGCACCGTTG  23

Template        60  .......................  82

Reverse primer  1    ACGAACCATCAAATCGCGAACCTA  24

Template        367  ........................  344
```

>KY963818.1 *Paracoccidioides brasiliensis* isolate T20B15-1 immunodominant antigen Gp43 (GP43) gene, exon 2 and partial cds

```
product length = 308

Forward primer  1   TCGTGATATAGACAGCACCGTTG  23

Template        60  .......................  82

Reverse primer  1    ACGAACCATCAAATCGCGAACCTA  24

Template        367  ........................  344
```

>KY963815.1 *Paracoccidioides brasiliensis* isolate 8652 immunodominant antigen Gp43 (GP43) gene, exon 2 and partial cds

```
product length = 308

Forward primer  1   TCGTGATATAGACAGCACCGTTG  23

Template        60  .......................  82

Reverse primer  1    ACGAACCATCAAATCGCGAACCTA  24

Template        367  ........................  344
```

>KY963814.1 *Paracoccidioides brasiliensis* isolate 3051 immunodominant antigen Gp43 (GP43) gene, exon 2 and partial cds

```
product length = 308

Forward primer  1   TCGTGATATAGACAGCACCGTTG  23

Template        60  .......................  82

Reverse primer  1    ACGAACCATCAAATCGCGAACCTA  24

Template        367  ........................  344
```

>KY963813.1 *Paracoccidioides brasiliensis* isolate T23B9-1 immunodominant antigen Gp43 (GP43) gene, exon 2 and partial cds

```
product length = 308

Forward primer  1   TCGTGATATAGACAGCACCGTTG  23

Template        60  .......................  82

Reverse primer  1    ACGAACCATCAAATCGCGAACCTA  24

Template        367  ........................  344
```

>KY963812.1 *Paracoccidioides brasiliensis* isolate T23LM1-1 immunodominant antigen Gp43 (GP43) gene, exon 2 and partial cds

```
product length = 308

Forward primer  1   TCGTGATATAGACAGCACCGTTG  23

Template        60  .......................  82

Reverse primer  1    ACGAACCATCAAATCGCGAACCTA  24

Template        367  ........................  344
```

>KY963811.1 *Paracoccidioides brasiliensis* isolate T22LM2-1 immunodominant antigen Gp43 (GP43) gene, exon 2 and partial cds

```
product length = 308

Forward primer  1   TCGTGATATAGACAGCACCGTTG  23

Template        60  .......................  82

Reverse primer  1    ACGAACCATCAAATCGCGAACCTA  24

Template        367  ........................  344
```

>KY963810.1 *Paracoccidioides brasiliensis* isolate T22LM1-1 immunodominant antigen Gp43 (GP43) gene, exon 2 and partial cds

```
product length = 308

Forward primer  1   TCGTGATATAGACAGCACCGTTG  23

Template        60  .......................  82

Reverse primer  1    ACGAACCATCAAATCGCGAACCTA  24

Template        367  ........................  344
```

>KY963809.1 *Paracoccidioides brasiliensis* isolate T20B13-1 immunodominant antigen Gp43 (GP43) gene, exon 2 and partial cds

```
product length = 308

Forward primer  1   TCGTGATATAGACAGCACCGTTG  23

Template        60  .......................  82

Reverse primer  1    ACGAACCATCAAATCGCGAACCTA  24

Template        367  ........................  344
```

>KY963808.1 *Paracoccidioides brasiliensis* isolate T19F33 immunodominant antigen Gp43 (GP43) gene, exon 2 and partial cds

```
product length = 308

Forward primer  1   TCGTGATATAGACAGCACCGTTG  23

Template        60  .......................  82

Reverse primer  1    ACGAACCATCAAATCGCGAACCTA  24

Template        367  ........................  344
```

>KY963807.1 *Paracoccidioides brasiliensis* isolate T19F24 immunodominant antigen Gp43 (GP43) gene, exon 2 and partial cds

```
product length = 308

Forward primer  1   TCGTGATATAGACAGCACCGTTG  23

Template        60  .......................  82

Reverse primer  1    ACGAACCATCAAATCGCGAACCTA  24

Template        367  ........................  344
```

>KY963806.1 *Paracoccidioides brasiliensis* isolate T19B15-2 immunodominant antigen Gp43 (GP43) gene, exon 2 and partial cds

```
product length = 308

Forward primer  1   TCGTGATATAGACAGCACCGTTG  23

Template        60  .......................  82

Reverse primer  1    ACGAACCATCAAATCGCGAACCTA  24

Template        367  ........................  344
```

>KY963805.1 *Paracoccidioides brasiliensis* isolate T19B7-2 immunodominant antigen Gp43 (GP43) gene, exon 2 and partial cds

```
product length = 308

Forward primer  1   TCGTGATATAGACAGCACCGTTG  23

Template        60  .......................  82

Reverse primer  1    ACGAACCATCAAATCGCGAACCTA  24

Template        367  ........................  344
```

>KY963804.1 *Paracoccidioides brasiliensis* isolate T18LM3-5 immunodominant antigen Gp43 (GP43) gene, exon 2 and partial cds

```
product length = 308

Forward primer  1   TCGTGATATAGACAGCACCGTTG  23

Template        60  .......................  82

Reverse primer  1    ACGAACCATCAAATCGCGAACCTA  24

Template        367  ........................  344
```

>KY963803.1 *Paracoccidioides brasiliensis* isolate T18LM3-3 immunodominant antigen Gp43 (GP43) gene, exon 2 and partial cds

```
product length = 308

Forward primer  1   TCGTGATATAGACAGCACCGTTG  23

Template        60  .......................  82

Reverse primer  1    ACGAACCATCAAATCGCGAACCTA  24

Template        367  ........................  344
```

>KY963802.1 *Paracoccidioides brasiliensis* isolate T18LM3-1 immunodominant antigen Gp43 (GP43) gene, exon 2 and partial cds

```
product length = 308

Forward primer  1   TCGTGATATAGACAGCACCGTTG  23

Template        60  .......................  82

Reverse primer  1    ACGAACCATCAAATCGCGAACCTA  24

Template        367  ........................  344
```

>KY963801.1 *Paracoccidioides brasiliensis* isolate T18LM1 immunodominant antigen Gp43 (GP43) gene, exon 2 and partial cds

```
product length = 308

Forward primer  1   TCGTGATATAGACAGCACCGTTG  23

Template        60  .......................  82

Reverse primer  1    ACGAACCATCAAATCGCGAACCTA  24

Template        367  ........................  344
```

>KY963800.1 *Paracoccidioides brasiliensis* isolate T17LM4 immunodominant antigen Gp43 (GP43) gene, exon 2 and partial cds

```
product length = 308

Forward primer  1   TCGTGATATAGACAGCACCGTTG  23

Template        60  .......................  82

Reverse primer  1    ACGAACCATCAAATCGCGAACCTA  24

Template        367  ........................  344
```

>KY963799.1 *Paracoccidioides brasiliensis* isolate T17LM3 immunodominant antigen Gp43 (GP43) gene, exon 2 and partial cds

```
product length = 308

Forward primer  1   TCGTGATATAGACAGCACCGTTG  23

Template        60  .......................  82

Reverse primer  1    ACGAACCATCAAATCGCGAACCTA  24

Template        367  ........................  344
```

>KY963798.1 *Paracoccidioides brasiliensis* isolate T17LM2 immunodominant antigen Gp43 (GP43) gene, exon 2 and partial cds

```
product length = 308

Forward primer  1   TCGTGATATAGACAGCACCGTTG  23

Template        60  .......................  82

Reverse primer  1    ACGAACCATCAAATCGCGAACCTA  24

Template        367  ........................  344
```

>KY656945.1 *Paracoccidioides brasiliensis* strain 46677 immunodominant antigen (gp43) gene, partial cds

```
product length = 308

Forward primer  1   TCGTGATATAGACAGCACCGTTG  23

Template        45  .......................  67

Reverse primer  1    ACGAACCATCAAATCGCGAACCTA  24

Template        352  ........................  329
```

>KY656944.1 *Paracoccidioides brasiliensis* strain 43659 immunodominant antigen (gp43) gene, partial cds

```
product length = 308

Forward primer  1   TCGTGATATAGACAGCACCGTTG  23

Template        44  .......................  66

Reverse primer  1    ACGAACCATCAAATCGCGAACCTA  24

Template        351  ........................  328
```

>KY656943.1 *Paracoccidioides brasiliensis* strain 43619 immunodominant antigen (gp43) gene, partial cds

```
product length = 308

Forward primer  1   TCGTGATATAGACAGCACCGTTG  23

Template        48  .......................  70

Reverse primer  1    ACGAACCATCAAATCGCGAACCTA  24

Template        355  ........................  332
```

>KY656942.1 *Paracoccidioides brasiliensis* strain 37550 immunodominant antigen (gp43) gene, partial cds

```
product length = 308

Forward primer  1   TCGTGATATAGACAGCACCGTTG  23

Template        45  .......................  67

Reverse primer  1    ACGAACCATCAAATCGCGAACCTA  24

Template        352  ........................  329
```

>KY656941.1 *Paracoccidioides brasiliensis* strain 29521 immunodominant antigen (gp43) gene, partial cds

```
product length = 308

Forward primer  1   TCGTGATATAGACAGCACCGTTG  23

Template        15  .......................  37

Reverse primer  1    ACGAACCATCAAATCGCGAACCTA  24

Template        322  ........................  299
```

>KX685516.1 *Paracoccidioides brasiliensis* isolate IPEC47735 immunodominant antigen gp43 gene, partial cds

```
product length = 308

Forward primer  1   TCGTGATATAGACAGCACCGTTG  23

Template        44  .......................  66

Reverse primer  1    ACGAACCATCAAATCGCGAACCTA  24

Template        351  ........................  328
```

>KX685515.1 *Paracoccidioides brasiliensis* isolate IPEC47003 immunodominant antigen gp43 gene, partial cds

```
product length = 308

Forward primer  1   TCGTGATATAGACAGCACCGTTG  23

Template        45  .......................  67

Reverse primer  1    ACGAACCATCAAATCGCGAACCTA  24

Template        352  ........................  329
```

>KX463654.1 *Paracoccidioides brasiliensis* strain IPEC30735 immunodominant antigen Gp43 (gp43) gene, partial cds

```
product length = 308

Forward primer  1   TCGTGATATAGACAGCACCGTTG  23

Template        45  .......................  67

Reverse primer  1    ACGAACCATCAAATCGCGAACCTA  24

Template        352  ........................  329
```

>KX463652.1 *Paracoccidioides brasiliensis* strain IPEC28455 immunodominant antigen Gp43 (gp43) gene, partial cds

```
product length = 308

Forward primer  1   TCGTGATATAGACAGCACCGTTG  23

Template        46  .......................  68

Reverse primer  1    ACGAACCATCAAATCGCGAACCTA  24

Template        353  ........................  330
```

>KX463650.1 *Paracoccidioides brasiliensis* strain IPEC27484 immunodominant antigen Gp43 (gp43) gene, partial cds

```
product length = 308

Forward primer  1   TCGTGATATAGACAGCACCGTTG  23

Template        46  .......................  68

Reverse primer  1    ACGAACCATCAAATCGCGAACCTA  24

Template        353  ........................  330
```

>KX463648.1 *Paracoccidioides brasiliensis* strain IPEC19346 immunodominant antigen Gp43 (gp43) gene, partial cds

```
product length = 308

Forward primer  1   TCGTGATATAGACAGCACCGTTG  23

Template        46  .......................  68

Reverse primer  1    ACGAACCATCAAATCGCGAACCTA  24

Template        353  ........................  330
```

>KU645890.1 *Paracoccidioides brasiliensis* strain IPEC20960 immunodominant antigen Gp43 gene, partial cds

```
product length = 308

Forward primer  1   TCGTGATATAGACAGCACCGTTG  23

Template        44  .......................  66

Reverse primer  1    ACGAACCATCAAATCGCGAACCTA  24

Template        351  ........................  328
```

>KU042924.1 *Paracoccidioides brasiliensis* strain IPEC47835 immunodominant antigen Gp43 gene, partial cds

```
product length = 308

Forward primer  1   TCGTGATATAGACAGCACCGTTG  23

Template        48  .......................  70

Reverse primer  1    ACGAACCATCAAATCGCGAACCTA  24

Template        355  ........................  332
```

>KT251016.1 *Paracoccidioides brasiliensis* strain EPM127 immunodominant antigen Gp43 (gp43) gene, partial cds

```
product length = 308

Forward primer  1   TCGTGATATAGACAGCACCGTTG  23

Template        20  .......................  42

Reverse primer  1    ACGAACCATCAAATCGCGAACCTA  24

Template        327  ........................  304
```

>KT251008.1 *Paracoccidioides brasiliensis* strain EPM54 immunodominant antigen Gp43 (gp43) gene, partial cds

```
product length = 308

Forward primer  1   TCGTGATATAGACAGCACCGTTG  23

Template        20  .......................  42

Reverse primer  1    ACGAACCATCAAATCGCGAACCTA  24

Template        327  ........................  304
```

>KT251007.1 *Paracoccidioides brasiliensis* strain EPM35 immunodominant antigen Gp43 (gp43) gene, partial cds

```
product length = 308

Forward primer  1   TCGTGATATAGACAGCACCGTTG  23

Template        20  .......................  42

Reverse primer  1    ACGAACCATCAAATCGCGAACCTA  24

Template        327  ........................  304
```

>KT251006.1 *Paracoccidioides brasiliensis* strain EPM29 immunodominant antigen Gp43 (gp43) gene, partial cds

```
product length = 308

Forward primer  1   TCGTGATATAGACAGCACCGTTG  23

Template        20  .......................  42

Reverse primer  1    ACGAACCATCAAATCGCGAACCTA  24

Template        327  ........................  304
```

>XM_010764774.1 *Paracoccidioides brasiliensis* Pb18 hypothetical protein partial mRNA

```
product length = 308

Forward primer  1    TCGTGATATAGACAGCACCGTTG  23

Template        693  .......................  715

Reverse primer  1     ACGAACCATCAAATCGCGAACCTA  24

Template        1000  ........................  977
```

>U26160.3 *Paracoccidioides brasiliensis* 43 kDa secreted glycoprotein precursor (gp43) gene, complete cds

```
product length = 308

Forward primer  1     TCGTGATATAGACAGCACCGTTG  23

Template        2818  .......................  2840

Reverse primer  1     ACGAACCATCAAATCGCGAACCTA  24

Template        3125  ........................  3102
```

>EU870211.1 *Paracoccidioides brasiliensis* isolate 769 immunodominant antigen Gp43 (gp43) gene, partial cds

```
product length = 308

Forward primer  1   TCGTGATATAGACAGCACCGTTG  23

Template        20  .......................  42

Reverse primer  1    ACGAACCATCAAATCGCGAACCTA  24

Template        327  ........................  304
```

>EU870210.1 *Paracoccidioides brasiliensis* isolate 133 immunodominant antigen Gp43 (gp43) gene, partial cds

```
product length = 308

Forward primer  1   TCGTGATATAGACAGCACCGTTG  23

Template        20  .......................  42

Reverse primer  1    ACGAACCATCAAATCGCGAACCTA  24

Template        327  ........................  304
```

>DQ768441.1 *Paracoccidioides brasiliensis* strain g14 43 kDa glycoprotein immunodominant antigen (GP43) gene, partial cds

product length = 308

Forward primer 1 TCGTGATATAGACAGCACCGTTG 23

Template 66 88

Reverse primer 1 ACGAACCATCAAATCGCGAACCTA 24

Template 373 350

>DQ768440.1 *Paracoccidioides brasiliensis* strain g11 43 kDa glycoprotein immunodominant antigen (GP43) gene, partial cds

product length = 308

Forward primer 1 TCGTGATATAGACAGCACCGTTG 23

Template 69 91

Reverse primer 1 ACGAACCATCAAATCGCGAACCTA 24

Template 376 353

>DQ768439.1 *Paracoccidioides brasiliensis* strain g9 43 kDa glycoprotein immunodominant antigen (GP43) gene, partial cds

product length = 308

Forward primer 1 TCGTGATATAGACAGCACCGTTG 23

Template 69 91

Reverse primer 1 ACGAACCATCAAATCGCGAACCTA 24

Template 376 353

>DQ768436.1 *Paracoccidioides brasiliensis* strain g7 43 kDa glycoprotein immunodominant antigen (GP43) gene, partial cds

product length = 308

Forward primer 1 TCGTGATATAGACAGCACCGTTG 23

Template 66 88

Reverse primer 1 ACGAACCATCAAATCGCGAACCTA 24

Template 373 350

>DQ768435.1 *Paracoccidioides brasiliensis* strain g2 43 kDa glycoprotein immunodominant antigen (GP43) gene, partial cds

product length = 308

Forward primer 1 TCGTGATATAGACAGCACCGTTG 23

Template 68 90

Reverse primer 1 ACGAACCATCAAATCGCGAACCTA 24

Template 375 352

>DQ768434.1 *Paracoccidioides brasiliensis* strain g1 43 kDa glycoprotein immunodominant antigen (GP43) gene, partial cds

product length = 308

Forward primer 1 TCGTGATATAGACAGCACCGTTG 23

Template 68 90

Reverse primer 1 ACGAACCATCAAATCGCGAACCTA 24

Template 375 352

>DQ768433.1 *Paracoccidioides brasiliensis* strain p166 43 kDa glycoprotein immunodominant antigen (GP43) gene, partial cds

product length = 308

Forward primer 1 TCGTGATATAGACAGCACCGTTG 23

Template 63 85

Reverse primer 1 ACGAACCATCAAATCGCGAACCTA 24

Template 370 347

>DQ768432.1 *Paracoccidioides brasiliensis* strain p165 43 kDa glycoprotein immunodominant antigen (GP43) gene, partial cds

```
product length = 308

Forward primer  1   TCGTGATATAGACAGCACCGTTG  23

Template        63  .......................  85

Reverse primer  1    ACGAACCATCAAATCGCGAACCTA  24

Template        370  ........................  347
```

>DQ003781.1 *Paracoccidioides brasiliensis* strain A4 immunodominant antigen Gp43 gene, partial cds

```
product length = 308

Forward primer  1   TCGTGATATAGACAGCACCGTTG  23

Template        60  .......................  82

Reverse primer  1    ACGAACCATCAAATCGCGAACCTA  24

Template        367  ........................  344
```

>DQ003773.1 *Paracoccidioides brasiliensis* strain V3 immunodominant antigen Gp43 gene, partial cds

```
product length = 308

Forward primer  1   TCGTGATATAGACAGCACCGTTG  23

Template        60  .......................  82

Reverse primer  1    ACGAACCATCAAATCGCGAACCTA  24

Template        367  ........................  344
```

>DQ003771.1 *Paracoccidioides brasiliensis* strain V1 immunodominant antigen Gp43 gene, partial cds

```
product length = 308

Forward primer  1   TCGTGATATAGACAGCACCGTTG  23

Template        60  .......................  82

Reverse primer  1    ACGAACCATCAAATCGCGAACCTA  24

Template        367  ........................  344
```

>DQ003750.1 *Paracoccidioides brasiliensis* strain C1 immunodominant antigen Gp43 gene, partial cds

```
product length = 308

Forward primer  1   TCGTGATATAGACAGCACCGTTG  23

Template        60  .......................  82

Reverse primer  1    ACGAACCATCAAATCGCGAACCTA  24

Template        367  ........................  344
```

>DQ003749.1 *Paracoccidioides brasiliensis* strain B26 immunodominant antigen Gp43 gene, partial cds

```
product length = 308

Forward primer  1   TCGTGATATAGACAGCACCGTTG  23

Template        60  .......................  82

Reverse primer  1    ACGAACCATCAAATCGCGAACCTA  24

Template        367  ........................  344
```

>DQ003744.1 *Paracoccidioides brasiliensis* strain B21 immunodominant antigen Gp43 gene, partial cds

```
product length = 308

Forward primer  1   TCGTGATATAGACAGCACCGTTG  23

Template        60  .......................  82

Reverse primer  1    ACGAACCATCAAATCGCGAACCTA  24

Template        367  ........................  344
```

>DQ003741.1 *Paracoccidioides brasiliensis* strain B18 immunodominant antigen Gp43 gene, partial cds

```
product length = 308

Forward primer  1   TCGTGATATAGACAGCACCGTTG  23

Template        60  .......................  82

Reverse primer  1    ACGAACCATCAAATCGCGAACCTA  24

Template        367  ........................  344
```

>DQ003736.1 *Paracoccidioides brasiliensis* strain B13 immunodominant antigen Gp43 gene, partial cds

```
product length = 308

Forward primer  1   TCGTGATATAGACAGCACCGTTG  23

Template        60  .......................  82

Reverse primer  1    ACGAACCATCAAATCGCGAACCTA  24

Template        367  ........................  344
```

>DQ003731.1 *Paracoccidioides brasiliensis* strain B8 immunodominant antigen Gp43 gene, partial cds

```
product length = 308

Forward primer  1   TCGTGATATAGACAGCACCGTTG  23

Template        60  .......................  82

Reverse primer  1    ACGAACCATCAAATCGCGAACCTA  24

Template        367  ........................  344
```

>DQ003730.1 *Paracoccidioides brasiliensis* strain B7 immunodominant antigen Gp43 gene, partial cds

```
product length = 308

Forward primer  1   TCGTGATATAGACAGCACCGTTG  23

Template        60  .......................  82

Reverse primer  1    ACGAACCATCAAATCGCGAACCTA  24

Template        367  ........................  344
```

>DQ003729.1 *Paracoccidioides brasiliensis* strain B6 immunodominant antigen Gp43 gene, partial cds

```
product length = 308

Forward primer  1   TCGTGATATAGACAGCACCGTTG  23

Template        60  .......................  82

Reverse primer  1    ACGAACCATCAAATCGCGAACCTA  24

Template        367  ........................  344
```

>DQ003724.1 *Paracoccidioides brasiliensis* strain B1 immunodominant antigen Gp43 gene, partial cds

```
product length = 308

Forward primer  1   TCGTGATATAGACAGCACCGTTG  23

Template        60  .......................  82

Reverse primer  1    ACGAACCATCAAATCGCGAACCTA  24

Template        367  ........................  344
```

>AY626381.1 *Paracoccidioides brasiliensis* strain Pb8 43 kDa secreted glycoprotein precursor, gene, partial cds

```
product length = 308

Forward primer  1   TCGTGATATAGACAGCACCGTTG  23

Template        90  .......................  112

Reverse primer  1    ACGAACCATCAAATCGCGAACCTA  24

Template        397  ........................  374
```

>AY626380.1 *Paracoccidioides brasiliensis* strain Pb4 43 kDa secreted glycoprotein precursor, gene, partial cds

```
product length = 308

Forward primer  1   TCGTGATATAGACAGCACCGTTG  23

Template        91  .......................  113

Reverse primer  1    ACGAACCATCAAATCGCGAACCTA  24

Template        398  ........................  375
```

>AY626379.1 *Paracoccidioides brasiliensis* strain Pb2 43 kDa secreted glycoprotein precursor, gene, partial cds

```
product length = 308

Forward primer  1   TCGTGATATAGACAGCACCGTTG  23

Template        91  .......................  113

Reverse primer  1    ACGAACCATCAAATCGCGAACCTA  24

Template        398  ........................  375
```

>AY626378.1 *Paracoccidioides brasiliensis* strain ATCC 60855 43 kDa secreted glycoprotein precursor, gene, partial cds

```
product length = 308

Forward primer  1   TCGTGATATAGACAGCACCGTTG  23

Template        60  .......................  82

Reverse primer  1    ACGAACCATCAAATCGCGAACCTA  24

Template        367  ........................  344
```

>AY626377.1 *Paracoccidioides brasiliensis* strain U1 43 kDa secreted glycoprotein precursor, gene, partial cds

```
product length = 308

Forward primer  1   TCGTGATATAGACAGCACCGTTG  23

Template        62  .......................  84

Reverse primer  1    ACGAACCATCAAATCGCGAACCTA  24

Template        369  ........................  346
```

>AY626376.1 *Paracoccidioides brasiliensis* strain P196 43 kDa secreted glycoprotein precursor, gene, partial cds

```
product length = 308

Forward primer  1   TCGTGATATAGACAGCACCGTTG  23

Template        61  .......................  83

Reverse primer  1    ACGAACCATCAAATCGCGAACCTA  24

Template        368  ........................  345
```

>AY619000.1 *Paracoccidioides brasiliensis* strain Cabbasous 43 kDa secreted glycoprotein precursor, gene, partial cds

```
product length = 308

Forward primer  1   TCGTGATATAGACAGCACCGTTG  23

Template        62  .......................  84

Reverse primer  1    ACGAACCATCAAATCGCGAACCTA  24

Template        369  ........................  346
```

>AY005437.1 *Paracoccidioides brasiliensis* clone B339 immunodominant antigen Gp43 (gp43) gene, complete cds

```
product length = 308

Forward primer  1    TCGTGATATAGACAGCACCGTTG  23

Template        771  .......................  793

Reverse primer  1     ACGAACCATCAAATCGCGAACCTA  24

Template        1078  ........................  1055
```

>AY005436.1 *Paracoccidioides brasiliensis* clone Arg-2 immunodominant antigen Gp43 (gp43) gene, complete cds

```
product length = 308

Forward primer  1    TCGTGATATAGACAGCACCGTTG  23

Template        771  .......................  793

Reverse primer  1     ACGAACCATCAAATCGCGAACCTA  24

Template        1078  ........................  1055
```

>AY005435.1 *Paracoccidioides brasiliensis* clone Arg-1 immunodominant antigen Gp43 (gp43) gene, complete cds

```
product length = 308

Forward primer  1    TCGTGATATAGACAGCACCGTTG  23

Template        771  .......................  793

Reverse primer  1     ACGAACCATCAAATCGCGAACCTA  24

Template        1078  ........................  1055
```

>AY005434.1 *Paracoccidioides brasiliensis* clone Ven-2 immunodominant antigen Gp43 (gp43) gene, complete cds

```
product length = 308

Forward primer  1    TCGTGATATAGACAGCACCGTTG  23

Template        771  .......................  793

Reverse primer  1     ACGAACCATCAAATCGCGAACCTA  24

Template        1078  ........................  1055
```

>AY005433.1 *Paracoccidioides brasiliensis* clone Ven-1 immunodominant antigen Gp43 (gp43) gene, complete cds

```
product length = 308

Forward primer  1    TCGTGATATAGACAGCACCGTTG  23

Template        771  .......................  793

Reverse primer  1     ACGAACCATCAAATCGCGAACCTA  24

Template        1078  ........................  1055
```

>AY005432.1 *Paracoccidioides brasiliensis* clone Solo-2 immunodominant antigen Gp43 (gp43) gene, complete cds

```
product length = 308

Forward primer  1    TCGTGATATAGACAGCACCGTTG  23

Template        771  .......................  793

Reverse primer  1     ACGAACCATCAAATCGCGAACCTA  24

Template        1078  ........................  1055
```

>AY005431.1 *Paracoccidioides brasiliensis* clone Solo-1 immunodominant antigen Gp43 (gp43) gene, complete cds

```
product length = 308

Forward primer  1    TCGTGATATAGACAGCACCGTTG  23

Template        771  .......................  793

Reverse primer  1     ACGAACCATCAAATCGCGAACCTA  24

Template        1078  ........................  1055
```

>AY005430.1 *Paracoccidioides brasiliensis* clone 18-2 immunodominant antigen Gp43 (gp43) gene, complete cds

```
product length = 308

Forward primer  1    TCGTGATATAGACAGCACCGTTG  23

Template        771  .......................  793

Reverse primer  1     ACGAACCATCAAATCGCGAACCTA  24

Template        1078  ........................  1055
```

>AY005428.1 *Paracoccidioides brasiliensis* clone 608-2 immunodominant antigen Gp43 (gp43) gene, complete cds

```
product length = 308

Forward primer  1    TCGTGATATAGACAGCACCGTTG  23

Template        771  .......................  793

Reverse primer  1     ACGAACCATCAAATCGCGAACCTA  24

Template        1078  ........................  1055
```

>AY005427.1 *Paracoccidioides brasiliensis* clone 608-1 immunodominant antigen Gp43 (gp43) pseudogene, complete sequence

```
product length = 308

Forward primer  1    TCGTGATATAGACAGCACCGTTG  23

Template        771  .......................  793

Reverse primer  1     ACGAACCATCAAATCGCGAACCTA  24

Template        1078  ........................  1055
```

>AY005425.1 *Paracoccidioides brasiliensis* clone SS-1 immunodominant antigen Gp43 (gp43) gene, complete cds

```
product length = 308

Forward primer  1    TCGTGATATAGACAGCACCGTTG  23

Template        771  .......................  793

Reverse primer  1     ACGAACCATCAAATCGCGAACCTA  24

Template        1078  ........................  1055
```

>AY005423.1 *Paracoccidioides brasiliensis* clone PRT1-1 immunodominant antigen Gp43 (gp43) gene, partial cds

```
product length = 308

Forward primer  1    TCGTGATATAGACAGCACCGTTG  23

Template        760  .......................  782

Reverse primer  1     ACGAACCATCAAATCGCGAACCTA  24

Template        1067  ........................  1044
```

>AY005419.1 *Paracoccidioides brasiliensis* clone Mg5-2 immunodominant antigen Gp43 (gp43) gene, complete cds

```
product length = 308

Forward primer  1    TCGTGATATAGACAGCACCGTTG  23

Template        771  .......................  793

Reverse primer  1     ACGAACCATCAAATCGCGAACCTA  24

Template        1078  ........................  1055
```

>AY005418.1 *Paracoccidioides brasiliensis* clone Mg5-1 immunodominant antigen Gp43 (gp43) gene, complete cds

```
product length = 308

Forward primer  1    TCGTGATATAGACAGCACCGTTG  23

Template        771  .......................  793

Reverse primer  1     ACGAACCATCAAATCGCGAACCTA  24

Template        1078  ........................  1055
```

>AY005417.1 *Paracoccidioides brasiliensis* clone Mg4-2 immunodominant antigen Gp43 (gp43) pseudogene, complete sequence

```
product length = 308

Forward primer  1    TCGTGATATAGACAGCACCGTTG  23

Template        771  .......................  793

Reverse primer  1     ACGAACCATCAAATCGCGAACCTA  24

Template        1078  ........................  1055
```

>AY005415.1 *Paracoccidioides brasiliensis* clone AP-2 immunodominant antigen Gp43 (gp43) gene, complete cds

```
product length = 308

Forward primer  1    TCGTGATATAGACAGCACCGTTG  23

Template        771  .......................  793

Reverse primer  1     ACGAACCATCAAATCGCGAACCTA  24

Template        1078  ........................  1055
```

>AY005414.1 *Paracoccidioides brasiliensis* clone AP-1 immunodominant antigen Gp43 (gp43) gene, complete cds

```
product length = 308

Forward primer  1    TCGTGATATAGACAGCACCGTTG  23

Template        771  .......................  793

Reverse primer  1     ACGAACCATCAAATCGCGAACCTA  24

Template        1078  ........................  1055
```

>AY005413.1 *Paracoccidioides brasiliensis* clone 9673-2 truncated immunodominant antigen Gp43 (gp43) gene, complete cds

```
product length = 308

Forward primer  1    TCGTGATATAGACAGCACCGTTG  23

Template        771  .......................  793

Reverse primer  1     ACGAACCATCAAATCGCGAACCTA  24

Template        1078  ........................  1055
```

>AY005412.1 *Paracoccidioides brasiliensis* clone 924-2 immunodominant antigen Gp43 (gp43) gene, complete cds

```
product length = 308

Forward primer  1    TCGTGATATAGACAGCACCGTTG  23

Template        771  .......................  793

Reverse primer  1     ACGAACCATCAAATCGCGAACCTA  24

Template        1078  ........................  1055
```

>AY005410.1 *Paracoccidioides brasiliensis* clone 470-2 immunodominant antigen Gp43 (gp43) gene, complete cds

```
product length = 308

Forward primer  1    TCGTGATATAGACAGCACCGTTG  23

Template        771  .......................  793

Reverse primer  1     ACGAACCATCAAATCGCGAACCTA  24

Template        1078  ........................  1055
```

>AY005409.1 *Paracoccidioides brasiliensis* clone 470-1 immunodominant antigen Gp43 (gp43) gene, complete cds

```
product length = 308

Forward primer  1    TCGTGATATAGACAGCACCGTTG  23

Template        771  .......................  793

Reverse primer  1     ACGAACCATCAAATCGCGAACCTA  24

Template        1078  ........................  1055
```

>AY005408.1 *Paracoccidioides brasiliensis* clone 1925-1 immunodominant antigen Gp43 (gp43) gene, complete cds

```
product length = 308

Forward primer  1    TCGTGATATAGACAGCACCGTTG  23

Template        771  .......................  793

Reverse primer  1     ACGAACCATCAAATCGCGAACCTA  24

Template        1078  ........................  1055
```

>AY005407.1 *Paracoccidioides brasiliensis* clone 1925-2 immunodominant antigen Gp43 (gp43) gene, complete cds

```
product length = 308

Forward primer  1    TCGTGATATAGACAGCACCGTTG  23

Template        771  .......................  793

Reverse primer  1     ACGAACCATCAAATCGCGAACCTA  24

Template        1078  ........................  1055
```

>AY005406.1 *Paracoccidioides brasiliensis* clone 1017-2 immunodominant antigen Gp43 (gp43) gene, complete cds

```
product length = 308

Forward primer  1    TCGTGATATAGACAGCACCGTTG  23

Template        771  .......................  793

Reverse primer  1     ACGAACCATCAAATCGCGAACCTA  24

Template        1078  ........................  1055
```

>AY005405.1 *Paracoccidioides brasiliensis* clone 1017-1 immunodominant antigen Gp43 (gp43) gene, complete cds

```
product length = 308

Forward primer  1    TCGTGATATAGACAGCACCGTTG  23

Template        771  .......................  793

Reverse primer  1     ACGAACCATCAAATCGCGAACCTA  24

Template        1078  ........................  1055
```

>MH029230.1 *Paracoccidioides brasiliensis* strain 30953 immunodominant antigen Gp43 (gp43) gene, partial cds

```
product length = 308

Forward primer  1  TCGTGATATAGACAGCACCGTTG  23

Template        1  -......................  22

Reverse primer  1    ACGAACCATCAAATCGCGAACCTA  24

Template        307  ........................  284
```

>AY005411.1 *Paracoccidioides brasiliensis* clone 924-1 immunodominant antigen Gp43 (gp43) gene, complete cds

```
product length = 308

Forward primer  1    TCGTGATATAGACAGCACCGTTG  23

Template        771  .............G.........  793

Reverse primer  1     ACGAACCATCAAATCGCGAACCTA  24

Template        1078  ........................  1055
```

Search parameter name	Search parameter value
Number of Blast hits analyzed	6181
Entrez query	
Min total mismatches	2
Min 3' end mismatches	2
Defined 3' end region length	5
Mismatch threshold to ignore targets	6
Max target size	4000
Max number of Blast target sequences	50000
Blast E value	30000
Blast word size	7
Max candidate primer pairs	500
Min PCR product size	66
Max PCR product size	1000
Min Primer size	15
Opt Primer size	20
Max Primer size	25
Min Tm	57
Opt Tm	60
Max Tm	63
Max Tm difference	3
Repeat filter	AUTO
Low complexity filter	Yes

Primer pair 2: *Paracoccidioides lutzii*

	Sequence (5'->3')	Length	Tm	GC%	Self complementarity	Self 3' complementarity
**Forward primer**	CTTCATGGCGCCCAAGGACTT	21	63.27	57.14	6.00	4.00
**Reverse primer**	GACTCCGCTAAGTCTGTCCTTAGGC	25	64.38	56.00	6.00	3.00

Products on target templates

>XM_002792442.2 *Paracoccidioides* lutzii Pb01 glucan 1,3-beta-glucosidase (PAAG_05770), partial mRNA

```
product length = 142

Forward primer  1    CTTCATGGCGCCCAAGGACTT  21

Template        762  .....................  782

Reverse primer  1    GACTCCGCTAAGTCTGTCCTTAGGC  25

Template        903  .........................  879
```

>JX065607.1 *Paracoccidioides* sp. 'lutzii' strain IEC-2005 immunodominant antigen Gp43 (gp43) gene, partial cds

```
product length = 142

Forward primer  1    CTTCATGGCGCCCAAGGACTT  21

Template        128  .....................  148

Reverse primer  1    GACTCCGCTAAGTCTGTCCTTAGGC  25

Template        269  .........................  245
```

>JN680102.1 *Paracoccidioides* sp. MS2 immunodominant antigen Gp43 (gp43) gene, partial cds

```
product length = 142

Forward primer  1   CTTCATGGCGCCCAAGGACTT  21

Template        89  .....................  109

Reverse primer  1    GACTCCGCTAAGTCTGTCCTTAGGC  25

Template        230  .........................  206
```

>JN680098.1 *Paracoccidioides* sp. MT-2011 strain ROSC immunodominant antigen Gp43 (gp43) gene, partial cds

```
product length = 142

Forward primer  1   CTTCATGGCGCCCAAGGACTT  21

Template        89  .....................  109

Reverse primer  1    GACTCCGCTAAGTCTGTCCTTAGGC  25

Template        230  .........................  206
```

>JN680092.1 *Paracoccidioides* sp. MT-2011 strain ED01 immunodominant antigen Gp43 (gp43) gene, partial cds

```
product length = 142

Forward primer  1   CTTCATGGCGCCCAAGGACTT  21

Template        89  .....................  109

Reverse primer  1    GACTCCGCTAAGTCTGTCCTTAGGC  25

Template        230  .........................  206
```

>EU870205.1 *Paracoccidioides brasiliensis* isolate 218 immunodominant antigen Gp43 (gp43) gene, partial cds

```
product length = 142

Forward primer  1   CTTCATGGCGCCCAAGGACTT  21

Template        89  .....................  109

Reverse primer  1    GACTCCGCTAAGTCTGTCCTTAGGC  25

Template        230  .........................  206
```

>EU870203.1 *Paracoccidioides brasiliensis* isolate 189 immunodominant antigen Gp43 (gp43) gene, partial cds

```
product length = 142

Forward primer  1   CTTCATGGCGCCCAAGGACTT  21

Template        89  .....................  109

Reverse primer  1    GACTCCGCTAAGTCTGTCCTTAGGC  25

Template        230  .........................  206
```

>EU870201.1 *Paracoccidioides brasiliensis* isolate 3171 immunodominant antigen Gp43 (gp43) gene, partial cds

```
product length = 142

Forward primer  1   CTTCATGGCGCCCAAGGACTT  21

Template        89  .....................  109

Reverse primer  1    GACTCCGCTAAGTCTGTCCTTAGGC  25

Template        230  .........................  206
```

>EU870199.1 *Paracoccidioides brasiliensis* isolate 6810 immunodominant antigen Gp43 (gp43) gene, partial cds

```
product length = 142

Forward primer  1   CTTCATGGCGCCCAAGGACTT  21

Template        89  .....................  109

Reverse primer  1    GACTCCGCTAAGTCTGTCCTTAGGC  25

Template        230  .........................  206
```

>EU870198.1 *Paracoccidioides brasiliensis* isolate 84 immunodominant antigen Gp43 (gp43) gene, partial cds

```
product length = 142

Forward primer  1   CTTCATGGCGCCCAAGGACTT  21

Template        89  .....................  109

Reverse primer  1    GACTCCGCTAAGTCTGTCCTTAGGC  25

Template        230  .........................  206
```

>EU870197.1 *Paracoccidioides brasiliensis* isolate 7455 immunodominant antigen Gp43 (gp43) gene, partial cds

```
product length = 142

Forward primer  1   CTTCATGGCGCCCAAGGACTT  21

Template        89  .....................  109

Reverse primer  1    GACTCCGCTAAGTCTGTCCTTAGGC  25

Template        230  .........................  206
```

>EU870196.1 *Paracoccidioides brasiliensis* isolate Pb01 immunodominant antigen Gp43 (gp43) gene, partial cds

```
product length = 142

Forward primer  1   CTTCATGGCGCCCAAGGACTT  21

Template        89  .....................  109

Reverse primer  1    GACTCCGCTAAGTCTGTCCTTAGGC  25

Template        230  .........................  206
```

>MT084540.1 *Lacazia loboi* isolate Bandino1 antigenic glycoprotein Gp43 gene, partial cds

```
product length = 139

Forward primer  1    CTTCATGGCGCCCAAGGACTT  21

Template        285  ..................G..  305

Reverse primer  1    GACTCCGCTAAGTCTGTCCTTAGGC  25

Template        423  A..C................G...  399
```

>EU109968.1 *Lacazia loboi* strain 17-LFC gp43-like protein gene, partial cds

```
product length = 139

Forward primer  1    CTTCATGGCGCCCAAGGACTT  21

Template        199  ..................G..  179

Reverse primer  1   GACTCCGCTAAGTCTGTCCTTAGGC  25

Template        61  A..C................G...  85
```

>EU109967.1 *Lacazia loboi* strain 6-SPS gp43-like protein gene, partial cds

```
product length = 139

Forward primer  1    CTTCATGGCGCCCAAGGACTT  21

Template        199  ..................G..  179

Reverse primer  1   GACTCCGCTAAGTCTGTCCTTAGGC  25

Template        61  A..C................G...  85
```

>EU109948.1 *Lacazia loboi* strain 12-S2 gp43-like protein gene, partial cds

```
product length = 139

Forward primer  1    CTTCATGGCGCCCAAGGACTT  21

Template        199  ..................G..  179

Reverse primer  1   GACTCCGCTAAGTCTGTCCTTAGGC  25

Template        61  A..C................G...  85
```

>EU109966.1 *Lacazia loboi* strain 13-FAV gp43-like protein gene, partial cds

```
product length = 139

Forward primer  1    CTTCATGGCGCCCAAGGACTT  21

Template        285  ..................G..  305

Reverse primer  1    GACTCCGCTAAGTCTGTCCTTAGGC  25

Template        423  A..C.................G...  399
```

>EU109965.1 *Lacazia loboi* strain 11-A gp43-like protein gene, partial cds

```
product length = 139

Forward primer  1    CTTCATGGCGCCCAAGGACTT  21

Template        285  ..................G..  305

Reverse primer  1    GACTCCGCTAAGTCTGTCCTTAGGC  25

Template        423  A..C.................G...  399
```

>EU109958.1 *Lacazia loboi* strain 4-RPM gp43-like protein gene, partial cds

```
product length = 139

Forward primer  1    CTTCATGGCGCCCAAGGACTT  21

Template        129  ..................G..  149

Reverse primer  1    GACTCCGCTAAGTCTGTCCTTAGGC  25

Template        267  A..C.................G...  243
```

>EU109957.1 *Lacazia loboi* strain 19-RCP gp43-like protein gene, partial cds

```
product length = 139

Forward primer  1    CTTCATGGCGCCCAAGGACTT  21

Template        285  ..................G..  305

Reverse primer  1    GACTCCGCTAAGTCTGTCCTTAGGC  25

Template        423  A..C.................G...  399
```

>EU109952.1 *Lacazia loboi* strain 33-RPGB gp43-like protein gene, partial cds

```
product length = 139

Forward primer  1    CTTCATGGCGCCCAAGGACTT  21

Template        285  ..................G..  305

Reverse primer  1    GACTCCGCTAAGTCTGTCCTTAGGC  25

Template        423  A..C.................G...  399
```

>EU109947.1 *Lacazia loboi* strain 10-RMS gp43-like protein gene, partial cds

```
product length = 139

Forward primer  1    CTTCATGGCGCCCAAGGACTT  21

Template        285  ..................G..  305

Reverse primer  1    GACTCCGCTAAGTCTGTCCTTAGGC  25

Template        423  A..C.................G...  399
```

>AY697436.1 *Lacazia loboi* gp43-like protein mRNA, partial cds

```
product length = 139

Forward primer  1    CTTCATGGCGCCCAAGGACTT  21

Template        285  ..................G..  305

Reverse primer  1    GACTCCGCTAAGTCTGTCCTTAGGC  25

Template        423  A..C.................G...  399
```

>EU109964.1 *Lacazia loboi* strain 14-BVS gp43-like protein gene, partial cds

```
product length = 139

Forward primer  1    CTTCATGGCGCCCAAGGACTT  21

Template        285  ..................G..  305

Reverse primer  1    GACTCCGCTAAGTCTGTCCTTAGGC  25

Template        423  A..C......G..........G...  399
```

Reference

1. Roberto, T.N.; Rodrigues, A.M.; Hahn, R.C.; de Camargo, Z.P. Identifying *Paracoccidioides* phylogenetic species by PCR-RFLP of the alpha-tubulin gene. *Med Mycol.* **2016**, *54*, 240-247, doi:10.1093/mmy/myv083.
2. Teixeira, M.M.; Theodoro, R.C.; de Carvalho, M.J.A.; Fernandes, L.; Paes, H.C.; Hahn, R.C.; Mendoza, L.; Bagagli, E.; San-Blas, G.; Felipe, M.S.S. Phylogenetic analysis reveals a high level of speciation in the *Paracoccidioides* genus. *Mol Phylogenet Evol* **2009**, *52*, 273-283, doi:http://dx.doi.org/10.1016/j.ympev.2009.04.005.
3. Marques-da-Silva, S.H.; Rodrigues, A.M.; de Hoog, G.S.; Silveira-Gomes, F.; de Camargo, Z.P. Occurrence of *Paracoccidioides lutzii* in the Amazon region: Description of two cases. *Am J Trop Med Hyg* **2012**, *87*, 710-714, doi:10.4269/ajtmh.2012.12-0340.
4. Hahn, R.C.; Rodrigues, A.M.; Fontes, C.J.; Nery, A.F.; Tadano, T.; de Padua Queiroz Junior, L.; de Camargo, Z.P. Fatal fungemia due to *Paracoccidioides lutzii*. *Am J Trop Med Hyg* **2014**, *91*, 394-398, doi:10.4269/ajtmh.13-0482.
5. Matute, D.R.; McEwen, J.G.; Puccia, R.; Montes, B.A.; San-Blas, G.; Bagagli, E.; Rauscher, J.T.; Restrepo, A.; Morais, F.; Niño-Vega, G., et al. Cryptic speciation and recombination in the fungus *Paracoccidioides brasiliensis* as revealed by gene genealogies. *Mol Biol Evol* **2006**, *23*, 65-73, doi:10.1093/molbev/msj008.
